# A central role for the transcriptional regulator VtlR in small RNA-mediated gene regulation in *Agrobacterium tumefaciens*

**DOI:** 10.1038/s41598-020-72117-0

**Published:** 2020-09-11

**Authors:** James A. Budnick, Lauren M. Sheehan, Miranda. J. Ginder, Kevin C. Failor, Julia. M. Perkowski, John. F. Pinto, Kirsten A. Kohl, Lin Kang, Pawel Michalak, Li Luo, Jason E. Heindl, Clayton C. Caswell

**Affiliations:** 1 Center for One Health Research, Virginia-Maryland College of Veterinary Medicine, Virginia Tech, Blacksburg, VA 24060 USA; 2 Department of Biological Sciences, University of the Sciences in Philadelphia, Philadelphia, PA 19104 USA; 3 Edward via College of Osteopathic Medicine, Blacksburg, VA 24060 USA; 4 Institute of Evolution, Haifa University, 3498838 Haifa, Israel; 5 Shanghai Key Laboratory of Bio-Energy Crops, School of Life Sciences, Plant Science Center, Shanghai University, Shanghai, 200444 China

**Keywords:** Bacterial genetics, Bacterial transcription, Bacteriology

## Abstract

LysR-type transcriptional regulators (LTTRs) are the most common type of transcriptional regulators in prokaryotes and function by altering gene expression in response to environmental stimuli. In the class *Alphaproteobacteria*, a conserved LTTR named VtlR is critical to the establishment of host-microbe interactions. In the mammalian pathogen *Brucella abortus*, VtlR is required for full virulence in a mouse model of infection, and VtlR activates the expression of *abcR2*, which encodes a small regulatory RNA (sRNA). In the plant symbiont *Sinorhizobium meliloti*, the ortholog of VtlR, named LsrB, is involved in the symbiosis of the bacterium with alfalfa. *Agrobacterium tumefaciens* is a close relative of both *B. abortus* and *S. meliloti*, and this bacterium is the causative agent of crown gall disease in plants. In the present study, we demonstrate that VtlR is involved in the ability of *A. tumefaciens* to grow appropriately in artificial medium, and an *A. tumefaciens vtlR* deletion strain is defective in motility, biofilm formation, and tumorigenesis of potato discs. RNA-sequencing analyses revealed that more than 250 genes are dysregulated in the ∆*vtlR* strain, and importantly, VtlR directly controls the expression of three sRNAs in *A. tumefaciens*. Taken together, these data support a model in which VtlR indirectly regulates hundreds of genes via manipulation of sRNA pathways in *A. tumefaciens*, and moreover, while the VtlR/LsrB protein is present and structurally conserved in many members of the *Alphaproteobacteria*, the VtlR/LsrB regulatory circuitry has diverged in order to accommodate the unique environmental niche of each organism.

## Introduction

LysR-type transcriptional regulators (LTTRs) are well represented in the three domains of life, and encompass the most common type of transcriptional regulator in prokaryotes^1^. First documented in 1988, this class of regulators can act as both activators and repressors of gene expression^2^. LTTRs are composed of two domains: a well-conserved N-terminal DNA-binding domain and a variable C-terminal substrate-binding domain.

The LTTR N-terminal domain is most commonly found as a helix-turn-helix, and regulated targets have a ‘classic’ LTTR-binding box sequence of TTA-N_7/8_-TAA^1^. The C-terminal domain is more variable among LTTRs. This domain can bind to a specific substrate and alter the activity of the protein. Some examples of substrate-sensing LTTRs include AphB from *Vibrio cholerae*^3–7^, BenM and CatM from *Acinetobacter baylyi*^8–10^, CbbR from *Rhodobacter sphaeroides*^11–13^, and OccR from *Agrobacterium tumefaciens*^14,15^. Alternatively, LTTRs can also undergo conformational changes without binding to a substrate. This is the case with OxyR, a redox-sensing LTTR responsible for activating genes important for responding to reactive oxygen species^16,17^. Overall, LTTRs play important regulatory roles in bacteria, allowing organisms to sense environmental cues and, in turn, swiftly alter gene expression through transcriptional activation and/or repression.

In the class *Alphaproteobacteria*, one highly conserved LTTR has been linked to efficient and effective host-bacterium interactions. First identified in the plant symbiont *Sinorhizobium meliloti*, the LTTR named LsrB (for **L**ysR-type **s**ymbiosis **r**egulator) is critical for the symbiosis of the bacterium and its host alfalfa (*Medicago sativa*)^18^. Regarding regulatory roles, *S. meliloti* LsrB is involved in the regulation of genes required for synthesizing glutathione and lipopolysaccharide^19,20^. Recently, *S. meliloti* LsrB was shown to have a similar sensing mechanism as OxyR, where the formation of intermolecular disulfide bonds in LsrB is involved in adaptation to oxidative stress, regulation of gene expression, proper alfalfa nodulation, and effective nitrogen fixation^21^.

An orthologous LTTR has also been characterized in the mammalian pathogen *Brucella abortus*^22^. In *B. abortus*, this LTTR, named VtlR (for **v**irulence-associated **t**ranscriptional **L**ysR-family **r**egulator), was shown to be a critical component in the ability of the bacterium to cause infection in both macrophages and mice. Microarray analysis revealed that *B. abortus* VtlR activates the expression of three genes encoding for small proteins. In addition, VtlR also positively regulates *abcR2*, encoding a sibling small regulatory RNA (sRNA) of the AbcR family. The AbcR sRNAs have been well documented to be involved in nutrient acquisition in the *Rhizobiales*^22–29^.

sRNAs are key regulatory components in bacteria and allow for rapid modification of gene expression, most commonly through post-transcriptional activation or repression of target mRNAs^30^. Two sRNAs found throughout the order *Rhizobiales* are the sibling AbcR sRNAs, AbcR1 and AbcR2^31^. The AbcR sRNAs regulate target mRNAs encoding ABC-type transport systems, many of which are responsible for transporting nutrients in specific environmental conditions^23–29^. Several of these mRNA targets encode transport systems found in *A. tumefaciens*, *S. meliloti* and *B. abortus*, and moreover, have been shown to be regulated by one or both of the AbcR sRNAs. However, it was unknown if the conservation of the AbcR system includes the transcriptional regulation of the sRNAs by VtlR/LsrB.

The present study aimed to characterize the LTTR VtlR in the plant pathogen *Agrobacterium tumefaciens*. Recently, it was reported that the VtlR ortholog in *A. tumefaciens* is required for efficient host-bacterium interactions, as well as other important processes, such as exopolysaccharide production, biofilm formation, and resistance to oxidative stress^32^. The present study confirms that VtlR is important for interactions between *A. tumefaciens* and plants, but this work also demonstrates that the significant global gene dysregulation observed in the *A. tumefaciens* ∆*vtlR* strain results predominantly from the direct activation of three small transcripts, all of which are authentic or purported small regulatory RNAs. Interestingly, we also demonstrate that LsrB is not involved in the expression of the AbcR sRNAs in *S. meliloti*, indicating that the VtlR/LsrB regulatory pathway has diverged significantly across members of the *Alphaproteobacteria*. Overall, the regulatory activity of VtlR differs dramatically from one bacterium to another, suggesting VtlR has evolved to fulfill the regulatory and environmental requirements of each particular bacterium.

## Results

### Expression of the sRNA AbcR1 is dependent on VtlR, and deletion of *vtlR* in *A. tumefaciens* results in a significant lag in growth in vitro

**Figure 1:**
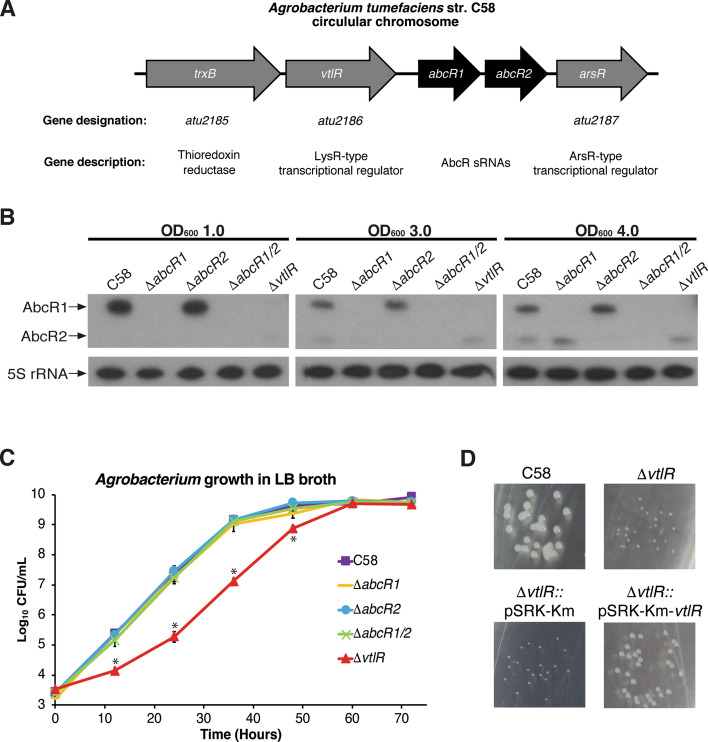
VtlR in *Agrobacterium tumefaciens* str. C58. (**A**) The *vtlR* gene (*atu2186*) is located on the *A. tumefaciens* circular chromosome, directly downstream of a thioredoxin reductase, *trxB* (*atu2185*). *vtlR* is upstream of the tandemly encoded AbcR sRNAs, *abcR1* and *abcR2*. Directly downstream of *abcR2* is an ArsR-type transcriptional regulator, *arsR* (*atu2187*). (**B**) Northern blot analyses for AbcR small RNAs. RNA was isolated from *A. tumefaciens* C58, ∆*vtlR*, ∆*abcR1*, ∆*abcR2*, and ∆*abcR1/2* cultures grown to optical densities (OD_600_) of 1.0, 3.0, or 4.0, separated on a denaturing polyacrylamide gels, transferred to nitrocellulose membranes, and probed with specific radiolabeled oligonucleotides. 5S ribosomal RNA was blotted for as a loading control. (**C**) In vitro growth kinetics of *A. tumefaciens* strains. The *A. tumefaciens* C58, *abcR1*, *abcR2*, *abcR1/2*, and *vtlR* strains were grown in LB broth, and at specified time points, samples from each culture were taken, serial diluted and plated on AT-agar plates to determine colony forming units (CFUs). Data represents average CFUs per mL ± the standard deviation of results from triplicate samples. (**D**) Photographs of *A. tumefaciens* colonies on ATGN + kanamycin (45 μg mL^−1^) agar after 72 h of growth.

The *vtlR* gene, designated as *atu2186,* is located on the circular chromosome of *A. tumefaciens* strain C58 (Fig. 1A). Directly upstream of *vtlR* is *trxB* (*atu2185*), a gene encoding a thioredoxin reductase; and downstream of *vtlR* are the genes encoding the AbcR sRNAs, *abcR1* and *abcR2*. It is interesting to note that the genetic organization of *trxB* and *vtlR* is well conserved in the class *Alphaproteobacteria*.

In *B. abortus*, VtlR has been shown to positively regulate the tandemly encoded sRNA *abcR2* on chromosome I; however the expression of *abcR1* on chromosome II of *B. abortus* is not regulated by VtlR^22^. Dissimilar from *B. abortus*, *abcR1* and *abcR2* are encoded directly downstream of *vtlR/lsrB* on the same chromosome in *A. tumefaciens* str. C58 (Fig. 1A) and *S. meliloti 1021*. It was unknown, however, if either of these sRNAs were regulated by the homolog of VtlR/LsrB (Atu2186 and SMc01225) in *A. tumefaciens* or *S. meliloti* respectively*.* To test whether VtlR regulated *abcR1* and/or *abcR2* in *A. tumefaciens*, northern blot analyses were performed to measure expression of AbcR1 and AbcR2 in wild-type *A. tumefaciens*, the isogenic deletion strains of *abcR1* (∆*abcR1*) and *abcR2* (∆*abcR2*), a double deletion strain of *abcR1 and abcR2* (∆*abcR1/2*)*,* and an isogenic deletion strain of *vtlR* (∆*vtlR*) (Fig. 1B). Bands representing the AbcR1 and AbcR2 transcripts were clearly visible when the *A. tumefaciens* culture is grown to an O.D. of 4.0 (Fig. 1B). Importantly, the AbcR1 and AbcR2 transcripts are not present in the isogenic deletion strains, confirming that the strains are in fact deletions of the indicated genes. Furthermore, the northern blot analyses also demonstrated that the expression level of AbcR2 is unaffected in ∆*vtlR*, but AbcR1 production is abolished in ∆*vtlR*. These data indicate that VtlR positively influences the expression of AbcR1, but not AbcR2, in *A. tumefaciens*. A similar strategy was employed to measure the expression of AbcR1 and AbcR2 in *S. meliloti 1021* and *S. meliloti 1021*:: ∆*lsrB*. Northern blot analysis revealed no change in the expression of the AbcRs in the absence of *lsrB*; indicating that *lsrB* does not regulate the expression of AbcR1 or AbcR2 in *S. meliloti* (Fig. S1).

In the plant symbiont *S. meliloti*, a deletion of *lsrB* resulted in a significant growth defect^18,20^; however, a deletion of *vtlR* in *B. abortus* showed no significant differences when grown in nutrient rich or nutrient limiting media^22^. *A. tumefaciens* str. C58, ∆*vtlR*, ∆*abcR1*, ∆*abcR2*, and ∆*abcR1/2* were grown in a nutrient-rich medium, and the number of colony-forming units (CFU) were measured every 12 h to examine growth over time in the deletion backgrounds to assess the necessity of *vtlR* for growth of *A. tumefaciens* (Fig. 1C). *A. tumefaciens* ∆*vtlR* displayed no difference in growth kinetics (i.e., doubling time during exponential growth) compared to the parental strain; however, similar to *S. meliloti*, *A. tumefaciens* ∆*vtlR* exhibited a lag in growth in liquid medium and small colony phenotype on agar medium when compared to the parental strain C58 (Fig. 1C,D). Isogenic deletions of *abcR1* and *abcR2*, and a double deletion of *abcR1* and *abcR2,* resulted in no differences in *A. tumefaciens* growth when compared to the parental strain (Fig. 1C). The lag in growth of the *A. tumefaciens* ∆*vtlR* was rescued by in-trans complementation with the plasmid pSRK-Km harboring an IPTG-inducible wild-type *vtlR* gene (Fig. 1D)^33^. As a control, *A. tumefaciens* ∆*vtlR* harboring an empty pSRK-Km plasmid showed no difference in growth compared to *A. tumefaciens* ∆*vtlR* (Fig. 1D). Altogether, these data reveal that VtlR positively regulates the sRNA AbcR1, and that VtlR is critical to the growth of *A. tumefaciens* in artificial medium.

### VtlR is critical to the virulence, biofilm formation, and motility of *A. tumefaciens* str. C58

**Figure 2:**
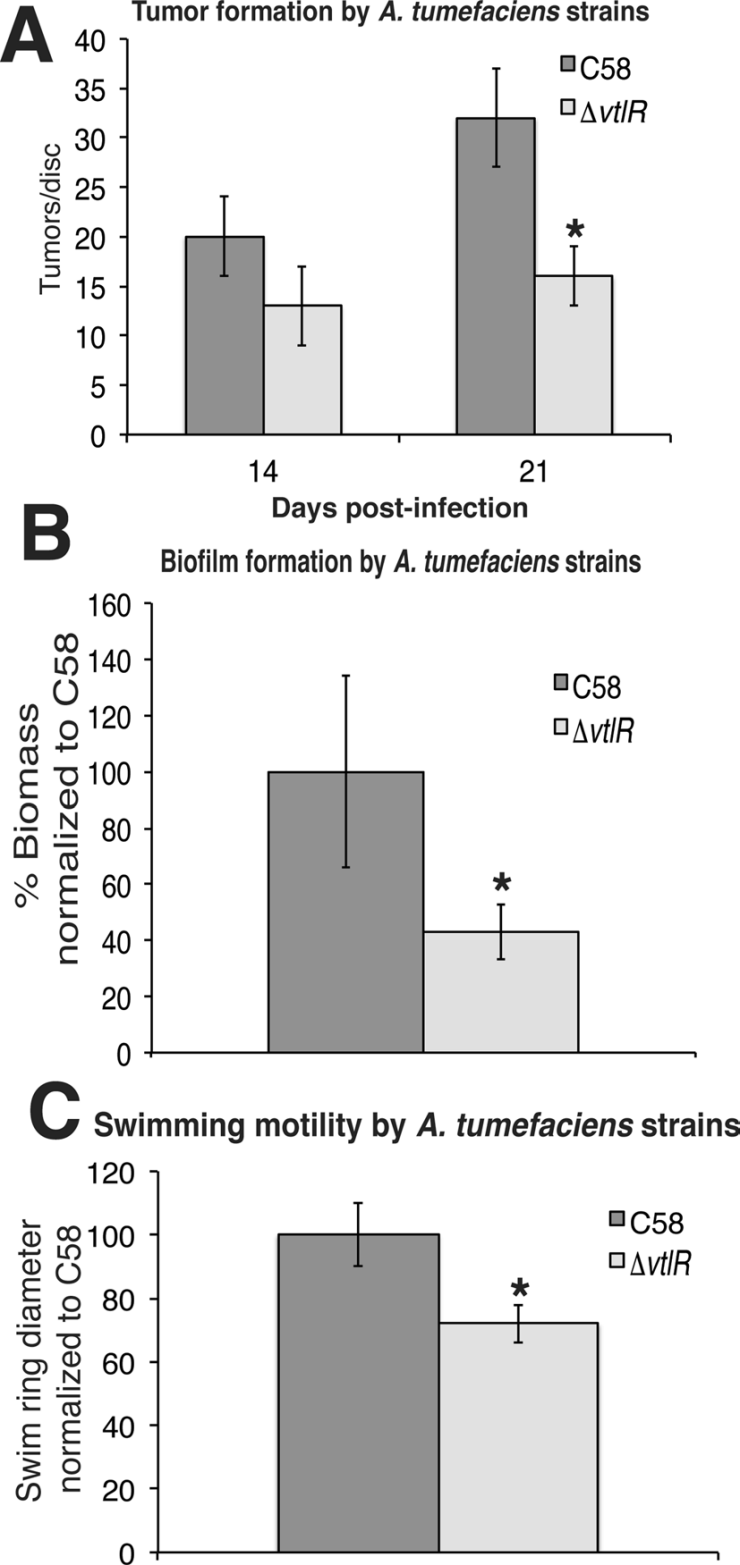
Contribution of *vtlR* to *A. tumefaciens*-mediated tumorigenesis, biofilm formation, and swimming motility. (**A**) Tumor formation of *A. tumefaciens* str. C58 and ∆*vtlR* in experimentally infected potatoes. Sterile potato discs were inoculated with C58 or ∆*vtlR*, and the number of tumors was counted 14- and 21-days post infection. Statistical significance is denoted by an asterisk (*) (*t* test; *P* < 0.05). (**B**) Biofilm formation by *A. tumefaciens* mutants. Data are means and standard deviations from three separate experiments normalized to C58. Static coverslip biofilm assays were performed as described and quantified after 48 h growth in LB. Statistical significance is denoted by an asterisk (*) (*t* test; *P* < 0.05). (**C**) Swim ring diameters were measured after single-colony inoculation into low density swim agar and incubation at room temperature for 7 days. The data are the mean of nine independent experiments. Statistical significance is denoted by an asterisk (*) (*t* test; *P* < 0.05).

VtlR was previously shown to be necessary for the symbiosis and pathogenesis of *S. meliloti* and *B. abortus*^18,22^. Moreover, recent work has demonstrated a role for VtlR in the pathogenesis of *A. tumefaciens* for attachment of the bacteria to the roots of *Arabidopsis* plants, as well as for the efficient transformation of tobacco leaves^32^. Since attachment and transformation are crucial virulence factors for the pathogenesis of *A. tumefaciens*, we hypothesized that *A. tumefaciens* ∆*vtlR* would also exhibit reduced tumorigenesis compared to the wild-type strain C58. To test this hypothesis, *A. tumefaciens* ∆*vtlR* was assessed for its ability to form tumors in experimentally infected potatoes (Fig. 2A). Compared to *A. tumefaciens* C58, the *vtlR* deletion strain caused, on average, the formation of fewer tumors 21 days post-infection (Fig. 2A). However, deletion of *abcR1*, *abcR2*, or a double deletion of *abcR1* and *abcR2* did not have any statistical difference in tumor formation compared to C58 (data not shown).

To further examine the function of VtlR in *A. tumefaciens*, biofilm production and swimming motility by ∆*vtlR* was assessed (Fig. 2B,C). *A. tumefaciens* ∆*abcR1* and ∆*abcR2* displayed no differences in biofilm production nor swimming motility when compared to the parental strain C58; however, the ∆*abcR1/2* strain displayed modest, but statistically insignificant, differences (data not shown). The *A. tumefaciens* ∆*vtlR* produced significantly less biofilm than the parental strain and displayed decreased swimming motility (Fig. 2B,C). Taken together, these data demonstrate that VtlR is important for the ability of *A. tumefaciens* to efficiently produce biofilms as well as sufficiently swim through agar, and this is consistent with previous work produced by Tang et al.^32^. However, it should be noted that it is possible that the growth defect exhibited by the *vtlR* deletion strain could be partly responsible for the observed decrease in motility, but the slower growth of ∆*vtlR* likely does not contribute to the decrease in biofilm formation, because the data are normalized to bacterial growth.

### The small RNA AbcR1 primarily regulates ABC-type transport systems

**Figure 3:**
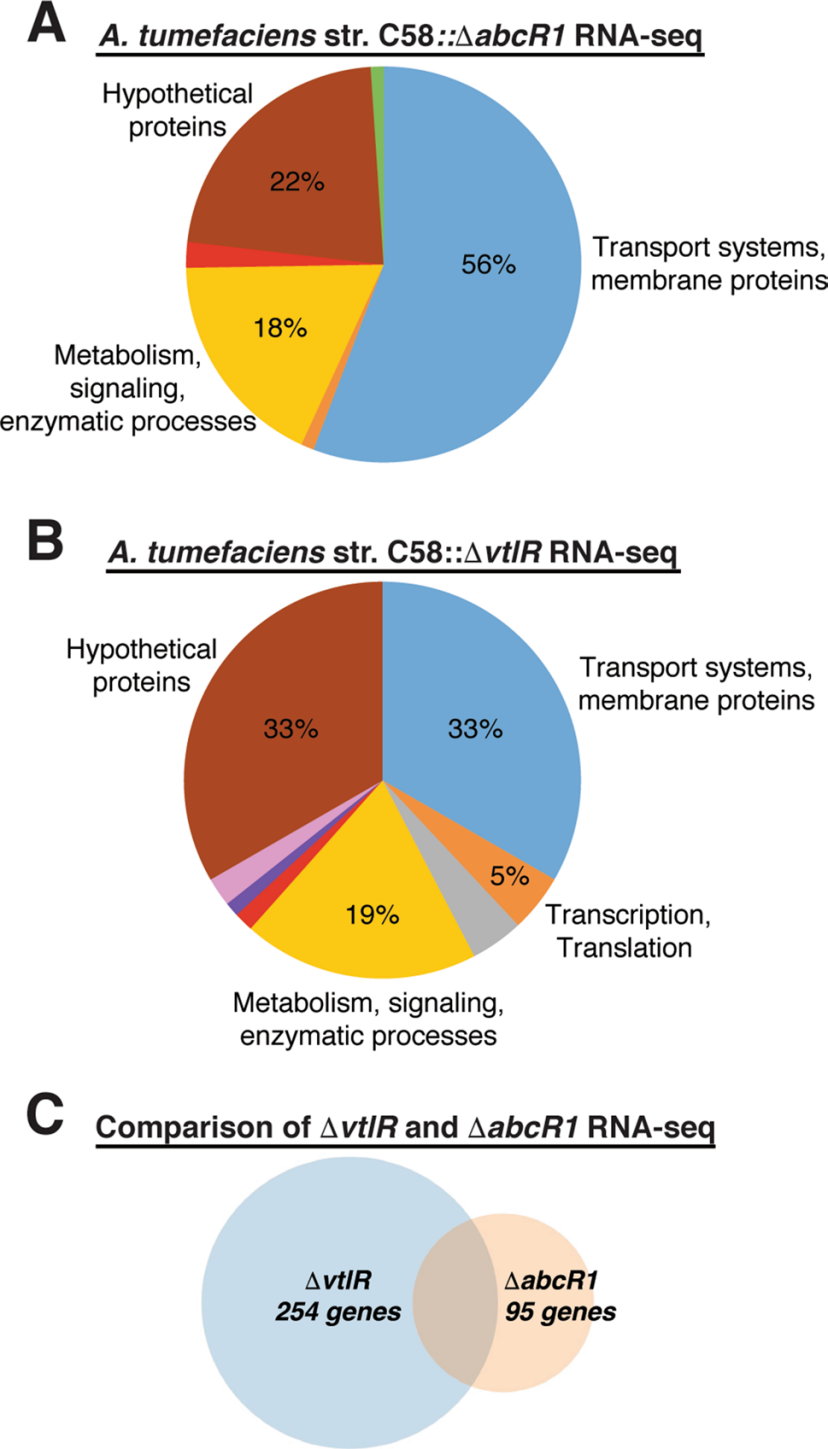
Overview of RNA-sequencing analyses of *A. tumefaciens* ∆*abcR1* and ∆*vtlR*. A. Pie chart summarizing genes differentially expressed (≥ 3-fold) in *A. tumefaciens* ∆*abcR1*. Blue: transport systems and membrane proteins (56%); brown: hypothetical proteins (22%); yellow: metabolism, signaling, enzymatic processes (18%); red: phage-related (2%); orange: transcription, translation (1%); green: chemotaxis, motility (1%). (**B**) Pie chart summarizing genes differentially expressed (≥ 3-fold) in *A. tumefaciens* ∆*vtlR*. Blue: transport systems and membrane proteins (33%); brown: hypothetical proteins (33%); yellow: metabolism, signaling, enzymatic processes (19%); orange: transcription, translation (5%); grey: secretion systems (4%); pink: polysaccharide biosynthesis (3%); red: phage-related (2%); purple: conjugation (1%). (**C**) Venn diagram comparing genes differentially expressed in *A. tumefaciens* ∆*vtlR* (254 genes) and *A. tumefaciens* ∆*abcR1* (95 genes) deletion strains.

To better understand the role of VtlR in *A. tumefaciens* pathogenesis, we sought next to define the transcriptional regulons of VtlR, AbcR1, and AbcR2. RNA-sequencing (RNA-seq) was carried out to compare RNA levels in ∆*vtlR*, ∆*abcR1*, and ∆*abcR2* to the parental strain C58 when cultured to late exponential phase in nutrient rich broth. Given that the *vtlR* deletion strain exhibits a growth defect compared to the other strains (Fig. 1C), RNA was isolated from cultures are the same OD_600_ (OD_600_ = 1.0) rather than a specific time point to ensure that the cells were in the same phase of growth. Additionally, the number of colony-forming units from each culture was also determined to confirm that the cultures contained similar numbers of viable bacteria. Although AbcR1 was dispensable for *A. tumefaciens* virulence, the regulon of AbcR1 is robust with almost 100 genes differentially expressed ≥ 3-fold in ∆*abcR1* when compared to the wild-type strain C58 (Supplementary Table S1). In comparison, only 8 genes were differentially expressed in ∆*abcR2*, and interestingly, half of the AbcR2-regulated genes encode hypothetical proteins (Supplementary Table S2). The significant difference in terms of the number of transcripts showing altered levels between AbcR1 over AbcR2 is in line with previous studies showing that AbcR1 exhibits more regulatory activity than AbcR2 in *Agrobacterium*^25,29^. Overall, the majority of genes differentially expressed in the *abcR1* deletion strain encode components of ABC-type transporters and membrane proteins (56%), followed by hypothetical proteins (22%), and genes involved in enzymatic processes (18%) (Fig. 3A). Many of these AbcR1 regulated genes have been confirmed by previously published proteomic analysis^25^.

### The *A. tumefaciens* VtlR transcriptional regulon is comprised of over 250 genes, including the AvhB type IV secretion system and a conjugal transfer system

RNA-seq analysis identified over 250 dysregulated genes in *A. tumefaciens* ∆*vtlR* when grown in LB broth (i.e., nutrient rich medium). Genes differentially expressed ≥ 3-fold in ∆*vtlR* are depicted in Supplementary Table S3, and qRT-PCR was utilized to validate differential expression of several of these targets in ∆*vtlR* (Table S4). An outline of the functional classification of the proteins encoded by the differentially expressed genes is shown in Fig. 3B. Of these dysregulated genes, 33% are predicted to encode membrane proteins and transport systems, 33% encode hypothetical proteins, and approximately 19% encode protein involved in metabolism, signaling, and enzymatic processes (Fig. 3B).

A comparison of the VtlR and AbcR1 regulons sheds light on the dependent and independent regulatory functions each possesses (Fig. 3C). For example, the VtlR regulon was found to be significantly larger than the AbcR1 regulon, with 254 dysregulated genes in ∆*vtlR* compared to 95 dysregulated genes in ∆*abcR1*. Approximately half of the genes differentially expressed ≥ 3-fold in *abcR1* were also differentially expressed in ∆*vtlR*. Altogether, these data reveal > 210 genes that could potentially be regulated by VtlR in an AbcR1-independent manner.

###  Agrobacterium tumefaciens VtlR directly activates the expression of *abcR1* and *atu1667*, a small hypothetical protein

**Figure 4:**
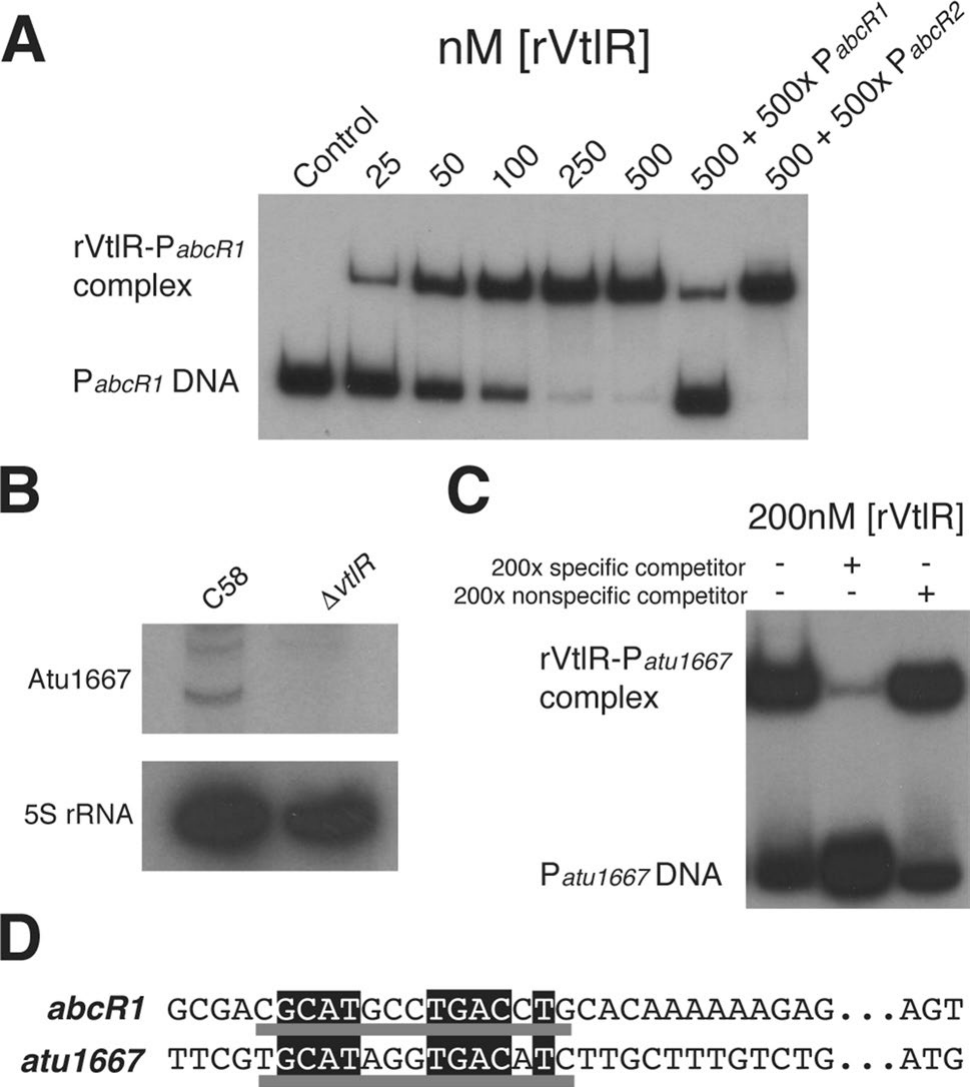
*A. tumefaciens* VtlR directly regulates *abcR1*, encoding a small RNA, and *atu1667*, encoding a small hypothetical protein. (**A**) Electrophoretic mobility shift assays (EMSAs) were carried out with recombinantly purified *A. tumefaciens* VtlR (rVtlR) and ^32^P-radiolabeled *abcR1* promoter region (P_*abcR1*_). Increasing concentrations of rVtlR were added to P_*abcR1*_, and the binding reactions were incubated at room temperature for 30 min. To determine specificity of binding, specific (unlabeled P_*abcR1*_) and non-specific (unlabeled P_*abcR2*_) competitors were added to some binding reactions. (**B**) Northern blot analysis confirming VtlR activation of *atu1667*. RNA from *A. tumefaciens* C58 and ∆*vtlR* was isolated from cultures grown in LB broth to OD_600_ 0.6, separated on a denaturing polyacrylamide gel, transferred to a nitrocellulose membrane, and probed with radiolabeled oligonucleotides. 5S ribosomal RNA was used as a loading control. (**C**) EMSAs were performed with rVtlR and ^32^P-radiolabeled *atu1667* promoter region (P_*atu1667*_). To determine specificity of binding, specific (unlabeled P_*atu1667*_) and non-specific (unlabeled P_*abcR2*_) competitors were added to some binding reactions. (**D**) Nucleotide alignment of the promoter regions of *abcR1* and *atu1667*. A consensus sequence is underlined in grey, with 100% identity of nucleotides highlighted in black.

To further characterize the regulatory mechanism of *A. tumefaciens* VtlR, electrophoretic mobility shift assays (EMSAs) were employed to test for the ability of recombinantly purified VtlR (rVtlR) to interact directly with the promoter regions of putative regulatory targets. As expected, VtlR bound to the promoter region on *abcR1* in a concentration-dependent manner (Fig. 4A). Moreover, the addition of unlabeled *abcR1* promoter region DNA competitively inhibited binding between rVtlR and the radiolabeled *abcR1* promoter, while excess unlabeled *abcR2* promoter DNA did not affect the formation of the rVtlR-P_*abcR1*_ binding complex. Overall, these data demonstrate that VtlR binds directly to the *abcR1* promoter region to activate *abcR1* expression in *A. tumefaciens*.

In *A. tumefaciens*, the gene *atu1667* encodes for a small hypothetical protein that is orthologous to BAB1_0914 and BAB2_0512 in *B. abortus*. *bab1_0914* and *bab2_0512* are directly transcriptionally activated by VtlR in *B. abortus*, and thus, it was hypothesized that VtlR also directly activates the expression of *atu1667* in *A. tumefaciens*^22^. To test this hypothesis, northern blot analysis was used to assess Atu1667 RNA levels in the *A. tumefaciens vtlR* deletion strain (Fig. 4B). Northern blot analysis and RNA-seq analysis (> 8-fold differentially expressed in ∆*vtlR*) showed that Atu1667 RNA levels were significantly decreased in *A. tumefaciens* ∆*vtlR* compared to the parental strain, suggesting that VtlR activates expression of *atu1667*. To assess potential binding between VtlR and the *atu1667* promoter region, EMSAs were performed with a radiolabeled promoter region of *atu1667* and rVtlR (Fig. 4C). These experiments determined that rVtlR binds directly and specifically to the promoter of *atu1667*, indicating that VtlR is a direct transcriptional activator of *atu1667* in *A. tumefaciens*.

### Identification of a VtlR-binding consensus sequence and discovery of a novel VtlR-regulated sRNA

**Figure 5:**
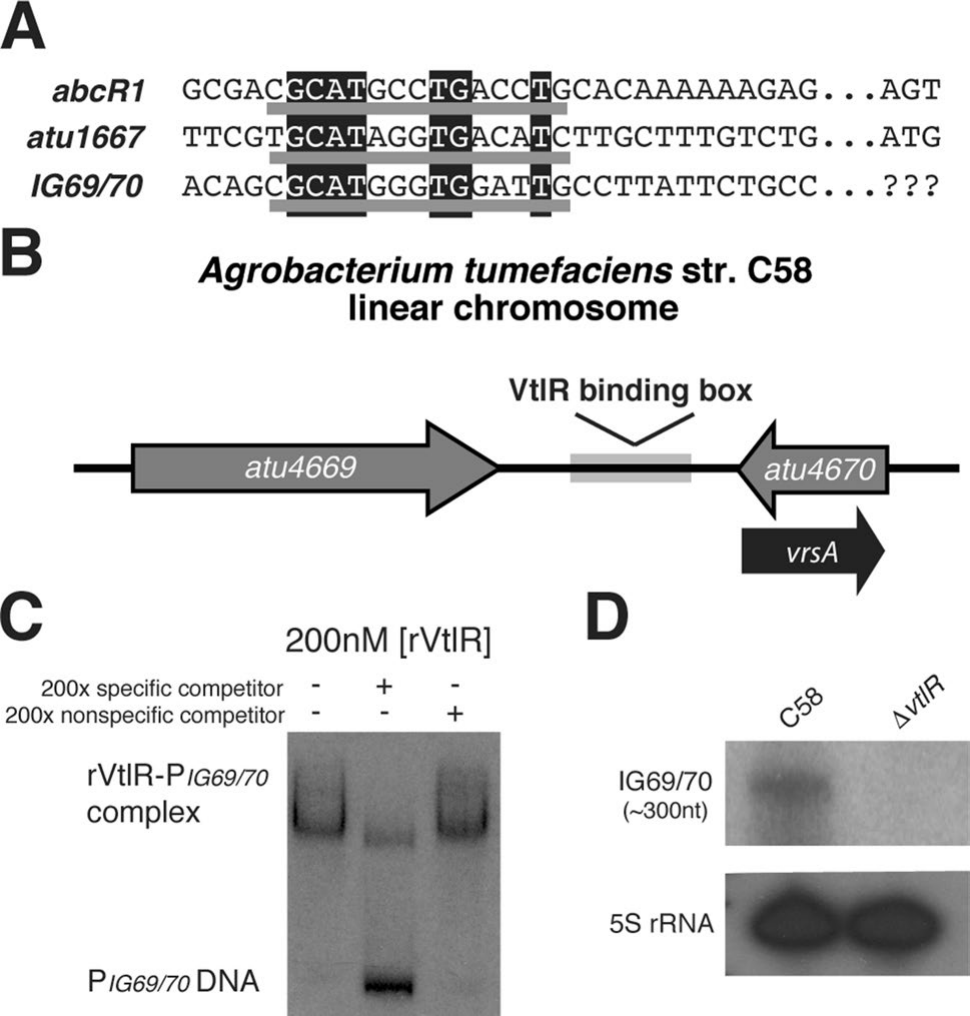
VtlR activates a novel transcript in *A. tumefaciens.* (**A**) Virtual footprinter revealed a novel VtlR-binding site in *A. tumefaciens* in the intergenic region of *atu4669* and *atu4670* (called *vrsA*)^34^. Nucleotide alignment of the promoter regions of *abcR1*, *atu1667*, and *vrsA* revealed a consensus sequence underlined in grey, with 100% identity of nucleotides highlighted in black. (**B**) The new VtlR-binding site is located on the linear chromosome in the intergenic region of two genes encoding for hypothetical proteins (*atu4669* and *atu4670*). The VtlR-binding box is depicted by a light grey box, and the putative transcript is depicted by the black arrow antisense to *atu4670*. (**C**) EMSAs were carried out with rVtlR and ^32^P-radiolabeled *vrsA* promoter region. To determine specificity of binding, specific (unlabeled P_*vrsA*_) and non-specific (unlabeled P_*abcR2*_) competitors were added to some binding reactions. (**D**) Northern blot analysis was employed to determine if VtlR regulates the putative transcript. RNA from *A. tumefaciens* C58 and ∆*vtlR* was isolated from cultures grown in LB broth to OD_600_ 0.6, separated on a denaturing polyacrylamide gel, transferred to a nitrocellulose membrane, and probed with radiolabeled oligonucleotides. 5S ribosomal RNA was used as a loading control.

A bioinformatic approach was employed to align the upstream regions of *abcR1* and *atu1667* and determine sequence similarities between the two promoters to identify a binding motif. Alignment of the two promoters led to the identification of a putative VtlR-binding sequence composed of 15 DNA base pairs (Fig. 4D). This putative VtlR consensus sequence was utilized to bioinformatically search for other potential VtlR binding sites in the *A. tumefaciens* genome. Putative VtlR sites were identified through the use of the online service Virtual Footprint^34^. Surprisingly, only one area displayed a match to the *abcR1/atu1667* VtlR-binding consensus sequence using this approach, and the identified area corresponds to an intergenic region flanked by *atu4669* and *atu4670* (Fig. 5A,B). Subsequently, an EMSA was performed with rVtlR and DNA that encompasses the *atu4669* and *atu4670* intergenic region (Fig. 5C). rVtlR bound directly and specifically to this intergenic region DNA.

Importantly, the promoter regions of *atu4669* and *atu4670* are not in this area of the chromosome; in contrast, this intergenic region is located between the 3′ ends of both *atu4669* and *atu4670* (Fig. 5B). Therefore, if VtlR is binding to a promoter region of a gene it regulates, then there may be a previously unannotated gene transcribed antisense to either *atu4669* or *atu4670*. To determine if an unannotated gene in encoded in this region, northern blot analysis was carried out with RNA isolated from C58 and ∆*vtlR.* Northern blot analysis revealed the presence of a ~ 300 nucleotide transcript antisense to *atu4670* (Fig. 5D). This small RNA was first identified as “L4” by Wilms et al. during a screen for previously unidentified sRNAs in the *Agrobacterium tumefaciens* genome^35^. The expression of this transcript was abolished in ∆*vtlR*. The evidence of direct binding of rVtlR to this DNA region and the decreased expression of the newly identified transcript in the *vtlR* deletion strain suggested that this new small RNA is under the direct transcriptional control of VtlR in *A. tumefaciens*. Thus, we have named this small RNA VrsA, for VtlR-regulated small RNA.

### The role of VtlR in sRNA regulation

Mentioned above, Wilms et al. utilized a differential RNA sequencing (dRNA-seq) strategy to identify novel sRNAs on all four of the *A. tumefaciens* replicons. They identified 228 new sRNAs in the *A. tumefaciens* genome via dRNA-seq and confirmed the existence of 22 of these sRNAs via northern blot analysis^35^. The ∆*vtlR* RNA-seq data was examined for differentially expressed sRNAs identified in the Wilms et al. dataset. Of the 228 sRNAs identified by Wilms et al., 24 sRNAs were predicted to be differentially expressed in *A. tumefaciens* ∆*vtlR* (Supplementary Table S5). Under the conditions tested, RNA was isolated from *A. tumefaciens* grown in LB broth to late exponential phase, only 7 of these sRNAs could be visualized via northern blot analysis and 2 were differentially expressed in *A. tumefaciens* ∆*vtlR* with restored expression in the complemented strain ∆*vtlR*::pSRK-Km-*vtlR*; one of which was the aforementioned VrsA (Supplementary Fig. S2). The second dysregulated sRNA in ∆*vtlR* is located within the intergenic region between *atu0985* and *atu0986* and is approximately 225 nucleotides in length; however, the proposed VtlR binding box is not found upstream of this new sRNA.

### VrsA does not contribute to the growth kinetics, tumor or biofilm formation, or motility of ***A. tumefaciens*** and 17 genes are differentially expressed in ∆***vrsA***

*Agrobacterium tumefaciens* ∆*abcR1* did not exhibit similar phenotypes observed in ∆*vtlR* and RNA-seq analysis showed that only a portion (~ 15%) of the genes dysregulated in ∆*vtlR* are potentially regulated in an AbcR1-dependent manner. With the discovery that VtlR also regulates the expression of *vrsA*, it is plausible that the > 210 AbcR1-independently regulated genes in ∆*vtlR* are regulated in a VrsA-dependent manner. To examine this hypothesis, an unmarked in-frame deletion of *atu4670*, the cis-encoded gene to *vrsA*, was constructed and utilized to perform phenotypic and RNA-seq analysis.

Phenotypic analyses were performed to assess growth, tumor and biofilm formation, and motility of *A. tumefaciens* ∆*vrsA* similarly to those performed in Figs. 1 and 2. *A. tumefaciens* ∆*vrsA* growth in nutrient rich medium, tumor and biofilm formation, and motility were not significantly different when compared to the parental strain *A. tumefaciens* str. C58 (Supplementary Fig. S3).

RNA was isolated from cultures of *A. tumefaciens* str. C58 and *A. tumefaciens* ∆*vrsA* grown to late exponential phase in LB broth and analyzed via RNA-seq analysis similarly to the transcriptomics performed in Supplementary Tables S1–S3. The data revealed 17 genes differentially expressed ≥ 3-fold in ∆*vrsA* compared to the parental strain C58 (Supplementary Table S6). Genes dysregulated in ∆*vrsA* are organized in 3 loci on the *A. tumefaciens* genome. One locus including genes encoding a putative transport system, *atu5126-atu5130*, was downregulated in the ∆*vrsA* RNA-seq dataset. Two loci, one including genes encoding a putative transport system and the other including genes involved in denitrification, were upregulated in ∆*vrsA* RNA-seq dataset.

It was possible that differences observed in gene expression were actually due to the loss of *atu4670* expression, rather than deletion of *vrsA*, because these genes are overlapping on the chromosome. To test this, we generated a strain harboring a mutated VtlR-binding box in the *vrsA* promoter that abolishes *vrsA* expression while not affecting the coding region of *atu4670* (Supplementary Fig. S4). Northern blot analyses revealed that the *vrsA*-SD strain produces no VrsA sRNA, and moreover, qRT-PCR analyses demonstrated that dysregulation of *att*, *agl*, and *nor* genes is similar between the ∆*vrsA* and *vrsA*-SD strains, indicating that disruption of *atu4670* is not responsible for the observed gene expression differences identified in the RNA-seq experiments with the ∆*vrsA* strain.

### Heterologous complementation of ***A. tumefaciens*** ∆***vtlR*** with ***S. meliloti lsrB*** or ***B. abortus vtlR***

**Figure 6:**
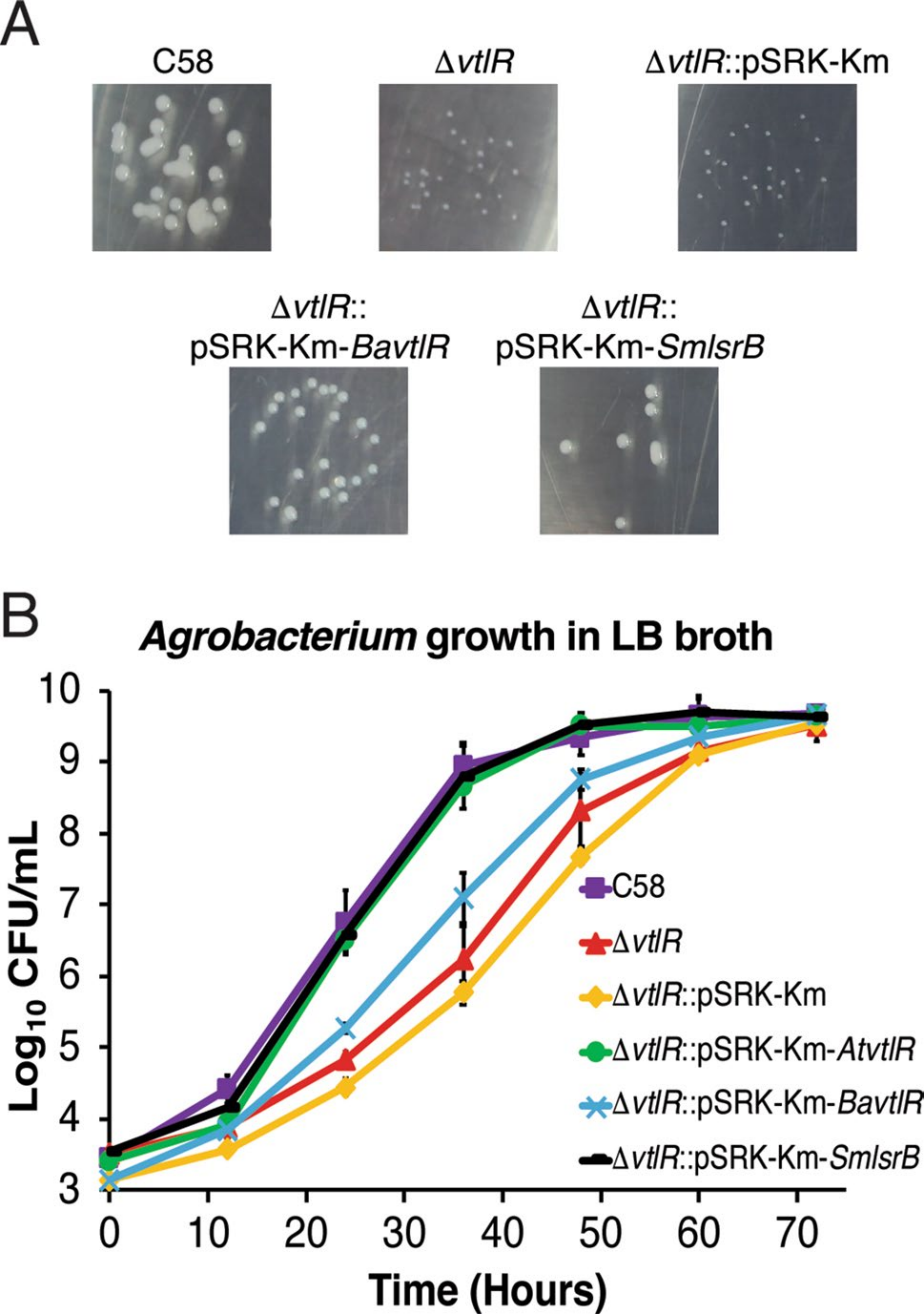
Heterologous complementation of *A. tumefaciens* ∆*vtlR* with *S. meliloti lsrB* and *B. abortus vtlR.* (**A**) Photographs of *A. tumefaciens* colonies on AT-agar after 72 h of growth. (**B**) The *A. tumefaciens* strains C58, ∆*vtlR*, and *vtlR*-complement strains were grown in LB broth with constant shaking at 28 °C. At 12-h intervals, samples from each culture were taken, serial diluted and plated out on AT-agar plates to determine colony-forming units (CFUs). Data represents average CFU per ml from each *A. tumefaciens* strain ± the standard deviation of results from triplicate samples. *Ba*, *Brucella abortus*; *Sm*, *Sinorhizobium meliloti.*

VtlR/LsrB proteins are highly conserved in many members of the *Rhizobiales*, and thus, it was hypothesized that *A. tumefaciens* ∆*vtlR* could be functionally complemented with a heterologous allele. The *A. tumefaciens* VtlR and *S. meliloti* LsrB, proteins share over 88% identity in amino acid sequence, whereas *A. tumefaciens* VtlR and *B. abortus* VtlR share 68% amino acid sequence identity. In *S. meliloti*, deletion of *lsrB* results in a severe growth defect in rich medium^18^. In contrast, there was no difference in the growth kinetics of *B. abortus* when *vtlR* was deleted^22^. To test our hypothesis, *vtlR* from *A. tumefaciens*, *vtlR* from *B. abortus,* and *lsrB* from *S. meliloti* were individually cloned into the expression vector pSRK-Km to complement the *A. tumefaciens vtlR* deletion strain, and the strains were assessed for growth in vitro. When plated on AT agar, wild-type colony size was restored with complementation of all three *vtlR* genes (Figs. 1D, 6A). This revealed the ability of *vtlR* from *A. tumefaciens*, *vtlR* from *B. abortus,* and *lsrB* from *S. meliloti* to heterologously complement the small colony phenotype of *A. tumefaciens vtlR*.

To assess the ability of VtlR orthologs to complement the *A. tumefaciens vtlR* growth defect in liquid medium, growth curves over time were conducted in rich medium supplemented with 100 μM IPTG and 45 μg mL^−1^ kanamycin (Fig. 6B). As a control, the growth defect displayed by *A. tumefaciens* ∆*vtlR* was not complemented by the presence of an empty pSRK-Km plasmid. *A. tumefaciens* ∆*vtlR* harboring the *lsrB* allele from *S. meliloti* or *vtlR* from *A. tumefaciens* restored wild-type growth kinetics (Fig. 6B). However, *vtlR* from *B. abortus* only partially restored growth of the *A. tumefaciens* ∆*vtlR*, revealing a potential divergence in function of *B. abortus* VtlR from other *Rhizobiales*.

## Discussion

In the present study, we have characterized a conserved LTTR named VtlR in the plant pathogen *A. tumefaciens* by defining its role in biological processes (i.e., tumorigenesis and biofilm formation) and genetic regulation (Fig. 1, 2, 3, 4, 5; Supplementary Table S3). Furthermore, this study revealed similarities and differences amongst three VtlR/LsrB orthologs from *A. tumefaciens*, *S. meliloti*, and *B. abortus* (Fig. 6). This work also further characterizes the regulatory capabilities of the AbcR sRNAs in *Agrobacterium* pathogenesis (Fig. 3; Supplementary Tables S1, S2), as well as a novel sRNA called VrsA (Supplementary Fig. S3; Supplementary Table S6).

The requirement of *vtlR* in *A. tumefaciens* pathogenesis resembles what was previously reported in two other *Rhizobiales*, *B. abortus* and *S. meliloti* (Fig. 2A)^18,22^. Moreover, our data support the observation that VtlR is important for host-bacterium interactions of *A. tumefaciens* with plants^32^. However, the dispensability of *abcR1* and *abcR2* in *A. tumefaciens* for efficient host-bacterium interactions and for proper growth of the bacteria differs from *S. meliloti* and *B. abortus*. In *S. meliloti*, deletion of *abcR1* or *abcR2* does not affect symbiosis, but a deletion of *abcR1* causes an acute growth defect in nutrient-rich medium^27^. In *B. abortus*, AbcR1 and AbcR2 are functionally redundant, and despite displaying no differences in bacterial growth in nutrient-rich or nutrient-limiting conditions, a strain containing a deletion of both *abcR1* and *abcR2* in *B. abortus* is less able to colonize and survive in macrophages and experimentally infected mice^24^. Conversely, AbcR1 and AbcR2 in *A. tumefaciens* are not functionally redundant. In *A. tumefaciens,* AbcR1 contains two RNA-binding motifs, named M1 (for motif 1) and M2 (for motif 2), and these motifs are utilized by AbcR1 to interact with target mRNAs^25^. The reason AbcR2 lacks robust regulatory functionality may have to do with the absence of M1 from its nucleotide sequence^31^. Altogether, data from this study supports previous work, where RNA-seq analysis found a deletion of *abcR1*, not *abcR2*, to lead to significant gene dysregulation (Fig. 3; Supplementary Tables S1, S2)^25^.

Although deletion of *abcR1* did not result in any phenotypic differences with regards to growth in vitro, virulence, or biofilm formation, we sought to further analyze the AbcR1 regulon (Fig. 3; Supplementary Table S1). RNA-seq analyses uncovered 95 differentially expressed genes (≥ 3-fold) in the *abcR1* deletion strain compared to the parental strain C58. Previously, Overlöper and colleagues utilized proteomic and bioinformatics analyses to characterize 16 targets of AbcR1, the majority of which are components of ABC-type transport systems (e.g., *chvE*, *malE*, *atu2422*, *atu4678*, and *atu1879*)^25^. Indeed, our transcriptomic data largely resembles the proteomic data from that study (Supplementary Table S1). However, aside from the ABC-type transport systems, RNA-seq revealed additional AbcR1 targets, such as genes encoding transcriptional regulators, chemoreceptors, and a variety of enzymes. Remarkably, several genes previously shown to be necessary for *A. tumefaciens* virulence, including *chvE* and *attC*, are dysregulated in ∆*abcR1* (Supplementary Table S1)^36,37^. Yet, a deletion of *abcR1* does not affect the ability of *A. tumefaciens* to form tumors in experimentally infected potatoes (Data not shown). A possible explanation for this may reside in the expression of these virulence-associated targets in ∆*abcR1*. While these target mRNAs exhibit decreased, but not completely abrogated, expression in ∆*abcR1*, it is possible that even the low level of expression of virulence-associated targets in ∆*abcR1* is sufficient to sustain infection, thus resulting in a lack of attenuation. Further investigation is needed to fully define the direct regulation and sRNA-mRNA interactions between AbcR1 and the newly identified targets in *A. tumefaciens*.

In *Agrobacterium*, VtlR is the transcriptional activator of *abcR1*, and directly binds to the promoter region of *abcR1* to exert its regulatory function (Figs. 1, 4). Following identification of gene dysregulation in both *A. tumefaciens* ∆*abcR1* and ∆*vtlR*, we sought to compare the transcriptomic profiles of the two deletion strains (Fig. 3). Importantly, several genetic systems differentially expressed in ∆*vtlR* showed no difference in *abcR1*, suggesting these systems could be key for *Agrobacterium* pathogenesis. Of note, a type IV secretion system (*avhB*), a conjugation system (*tra*), genes necessary for polysaccharide biosynthesis (*exo*), as well as 10 transcriptional regulators are all differentially expressed in ∆*vtlR* and show no difference in expression in ∆*abcR1*. However, none of these genes has been shown to contribute to or is associated with the virulence of *A. tumefaciens*^38^. With regards to biofilm production, one gene, *divK*, may contribute to differences in biofilm production observed in ∆*vtlR* (Fig. 2B). A deletion of *divK* has been reported to result in decreased biofilm production in *Agrobacterium*^39^. In *A. tumefaciens* ∆*vtlR*, *divK* is over-expressed, suggesting the possibility of *divK* dysregulation could alter biofilm formation. While no other genes from the ∆*vtlR* RNA-seq analysis have been linked directly to biofilm formation, several genes have been speculated to be necessary for biofilm production (e.g., *glcF* and *gguB*)^40^.

The difference in size of the VtlR regulons in *Brucella* and *Agrobacterium* is striking. Indeed, the *B. abortus* VtlR regulon is comprised of 10 genes, while the *A. tumefaciens* VtlR regulon is comprised of > 200 genes (Supplementary Table S3)^22^. The complete regulon of LsrB in *S. meliloti* is currently unknown, although several genes involved in LPS synthesis, glutathione synthesis, and oxidative stress are regulated by LsrB^19,20^. Since *S. meliloti* LsrB was shown to directly bind to the promoter region of the *lrp3-lpsCDE* operon, it was hypothesized that the homologous *lrp3-lpsCDE* system in *A. tumefaciens* may be similarly regulated^20^. However, EMSAs showed no binding of *A. tumefaciens* rVtlR to the promoter region of *lrp3-lpsCDE* (Supplementary Fig. S5). Furthermore, the VtlR/LsrB systems have diverged in their regulation of the *abcR* sRNAs between organisms, as *S. meliloti* LsrB does not regulate the *abcR* sRNAs under the conditions tested (Supplementary Fig. S1). Regarding glutathione production, Tang et al. demonstrated that the genes *gshA* and *gshB* are regulated by OxyR^20^. OxyR, a LTTR responsible for aiding the cell in responding to oxidative stress, is transcriptionally activated by LsrB in *S. meliloti*^20^. Similar to *S. meliloti*, *A. tumefaciens* OxyR is necessary for protection against oxidative stress and important for host-microbe interactions^41,42^. In contrast to *S. meliloti*, *oxyR* does not appear to be regulated by VtlR in *A. tumefaciens*, as no difference in *oxyR* expression in *A. tumefaciens* ∆*vtlR* was found (Supplementary Table S3). Overall, these findings suggest a divergence of function between *S. meliloti* LsrB and *A. tumefaciens* VtlR.

A bioinformatics approach led to the identification of a novel sRNA regulated by VtlR in *A. tumefaciens* by searching the *A. tumefaciens* genome for additional VtlR binding sites (Fig. 5A)^34^. Initially, EMSA analyses were performed with rVtlR and the promoter regions of genes exhibiting significantly differential expression in ∆*vtlR* (Supplementary Table S3), including promoters from the following genes: *atu0036*, *atu0055*, *atu0157*, *atu0323*, *atu0463*, *atu2708*, *atu3939*, *atu4669*, *atu5116*, *atu5119*, *atu5121*, *atu5161*, *atu5118*, *atu0484*, *atu0828*, *atu1296*, *atu2187*, *atu2350*, *atu2384*, *atu3252*, *atu4782*, *atrA*, *atrB*, *avhB1*, and *chvE*. Surprisingly, we determined that the *A. tumefaciens* rVtlR protein did not interact with any of these promoter regions under the conditions tested, aside from *abcR1* (data not shown).

As mentioned in the introduction, the N-terminal domain LysR-type transcriptional regulators (LTTRs) most commonly include a helix–turn–helix domain, and regulated targets have a ‘classic’ LTTR-binding box sequence of TTA-N_7/8_-TAA, in an A/T rich region^1^. Thus, the proposed VtlR binding box confirmed in *B. abortus* and hypothesized in *A. tumefaciens* of GCAT-N_3_-TG-N_3_-T is divergent from typical LTTRs and may be unique to this regulator among the *Rhizobiales*.

Identification of a putative VtlR binding box, Fig. 4D, and genome-wide search of this sequence in *A. tumefaciens* identified a region upstream of a novel sRNA previously described as “L4” but has been renamed VrsA (VtlR-regulated sRNA)^35^. Our data shows that VrsA is directly activated by VtlR (Fig. 5). Phenotypic and transcriptional analyses described in this study revealed that a deletion of *vrsA* does not contribute to the growth, tumor or biofilm formation, or motility of *A. tumefaciens* (Supplementary Fig. S3). RNA-seq analysis revealed that 17 genes were differentially expressed in *A. tumefaciens* ∆*vrsA* grown in nutrient rich broth (Supplementary Table S6). The *att* operon (i.e., *atu5126-atu5130*) encoding a transport and attachment system was down-regulated in the ∆*vrsA* strain, indicating that VrsA activates the expression of these genes^37^. This supports the VtlR regulatory model because *att* also had lowered expression in ∆*vtlR*. Thus, VtlR activates the expression of *vrsA*, which in turn activates the expression of *att*. The expression of two loci were increased in ∆*vrsA*, and these genes include a putative transport system, *atu0591–atu0593*, and the *nor* operon, which encodes a nitric oxide reductase important for denitrification. Overall, further mechanistic analyses are necessary to understand the regulatory role of the VrsA sRNA on these targets.

Two other small RNA encoding genes were differentially expressed in ∆*vtlR*. *atu1667* encodes a putative hypothetical protein that has not yet been characterized in *Agrobacterium,* but BAB2_0512, a homolog of Atu1667, encodes a small protein that is linked to the ability of *B. abortus* to utilize the sugar fucose^43^. It is unknown whether *atu1667* also encodes a small protein or if it potentially functions as a small regulatory RNA. Northern blot analysis also revealed the presence of a small RNA in the intergenic region of *atu0985* and *atu0986* that is activated by VtlR (Supplementary Table S5; Supplementary Fig. S2). However, the VtlR binding-box is not found in this region, and as such, it is not known if VtlR directly or indirectly regulates this transcript nor if this transcript has downstream regulatory functions.

A recent study described the necessity of VtlR in *A. tumefaciens* host–bacterium interactions^32^. There are several similarities observed between our work and the study by Tang et al., but the studies also contain differences with regards to experimental approach and results observed. One major difference is the *Agrobacterium* strain utilized. The work presented here employed *A. tumefaciens* str. C58, which is a naturally occurring strain of *A. tumefaciens*, whereas Tang et al. utilized a strain named *A. tumefaciens* C58C1^32^. *A. tumefaciens* C58C1 is a modified *A. tumefaciens* strain in which the original virulence plasmid pTiC58 is replaced by the *A. rhizogenes* virulence plasmid, pRiA4b^44^. While the C58C1 strain is still able to form host-microbe interactions and cause disease in its plant host, this absence may affect the function of VtlR in *A. tumefaciens*.

While there are many similarities in gene expression when comparing the present RNA-seq dataset to the previously published ∆*vtlR* dataset, there are some significant differences (Supplementary Table S3)^32^. It should be noted that between the studies, *A. tumefaciens* strains were grown in different medium. In the presented study, RNA was isolated from *A. tumefaciens* grown in LB broth, whereas the previous study isolated RNA from bacteria grown in TY broth (Supplementary Table S3)^32^. The largest difference between the VtlR regulons from these two studies is the differential gene expression observed with regards to AbcR1. Our data and data previously presented by Overlöper and colleagues show differences in expression and regulation of *chvE*, *atu2422*, *atu4678*, and *atu1879* in ∆*abcR1* and ∆*vtlR* (Supplementary Tables S1, S3)^25^. None of these genes appear to be differentially regulated in the VtlR regulon described previously^32^. Altogether, these differences may be explained by the variation in experimental design, particularly in regards to the conditions under which the bacteria were cultured before RNA isolation.

**Figure 7:**
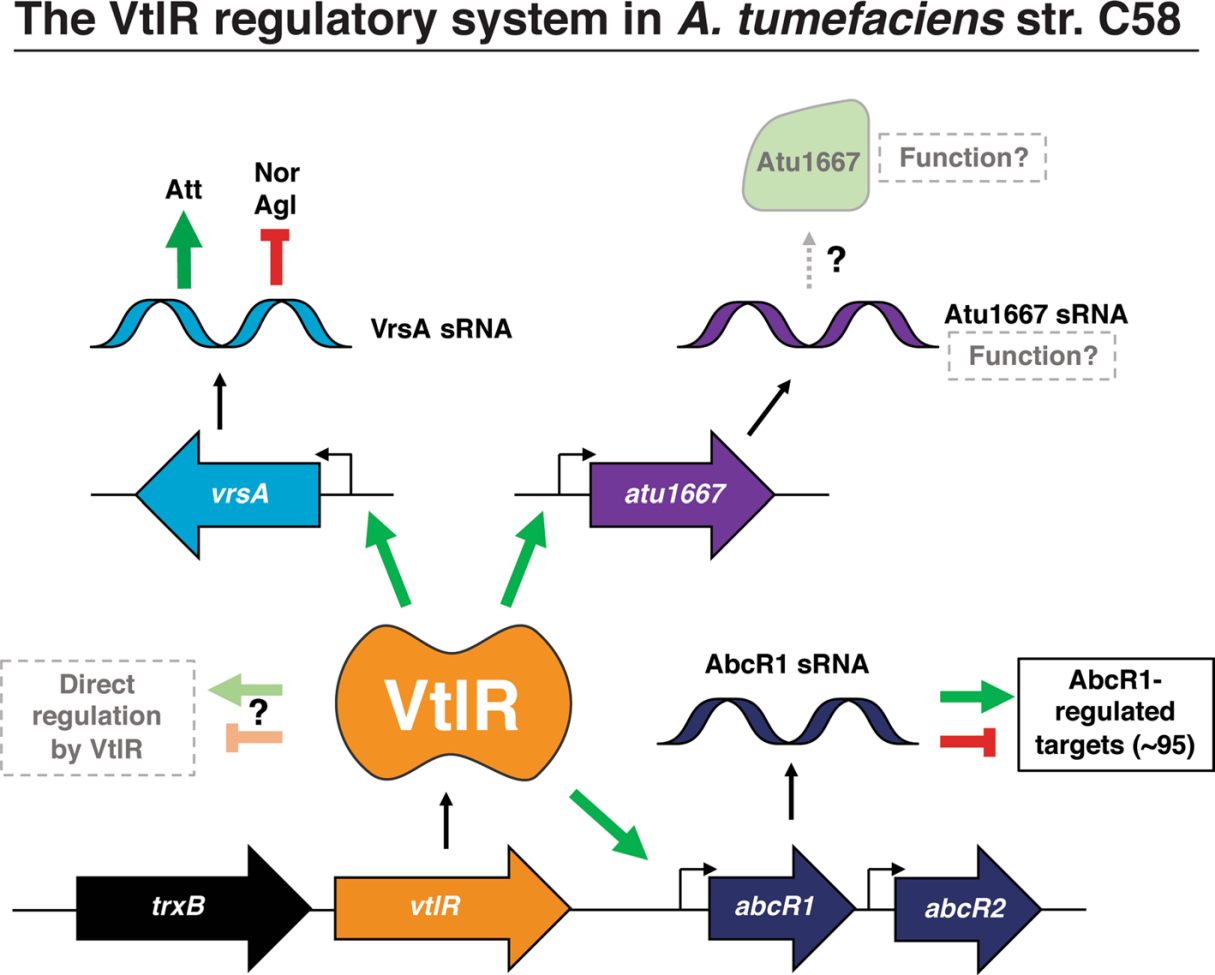
Working model of VtlR regulation in *A. tumefaciens*. *vtlR* (orange) is located downstream of the thioredoxin reductase, *trxB* (black) and upstream of the tandemly encoded sRNAs, *abcR1* and *abcR2*. VtlR directly activates *abcR1*, *vrsA* (blue) and *atu1667* (purple). AbcR1 regulates ~ 95 targets independent of VtlR, while VrsA regulates ~ 15 targets independent of VtlR. The regulatory capacity of Atu1667 is currently unknown.

In conclusion, VtlR is necessary for efficient in vitro growth and tumorigenesis of the plant pathogen *A. tumefaciens*. The *A. tumefaciens* VtlR protein binds directly to a conserved binding box in three promoter regions, but VtlR is linked to the regulation of over 250 genes. Taken together, we propose a model of regulation by VtlR in *A. tumefaciens* in which VtlR plays a central role in the activation of sRNAs that, in turn, control the expression of a wide variety of mRNA targets (Fig. 7). In this model, VtlR activates the expression of AbcR1, the newly described sRNA, VrsA, and the putative sRNA Atu1667, and these sRNAs are the major regulatory elements responsible for propagating the VtlR genetic circuit. Overall, this study demonstrates the functional importance of VtlR in *A. tumefaciens*, and provides insight into the evolutionary similarities and differences that exist in the VtlR/LsrB systems of members of the *Rhizobiales*.

## Methods

### Bacterial strains and growth conditions

*Agrobacterium tumefaciens* str. C58 and derivative strains were routinely grown on Luria–Bertani (LB) agar (Fisher Scientific Company LLC, Suwanee, GA) and cultures were routinely grown in LB broth. LB consists of 10 g NaCl, 10 g Bacto™-tryptone, and 5 g yeast extract per liter. For some experiments, *Agrobacterium* strains were grown in ATGN medium (AT minimal medium [79 mM KH_2_PO_4_, 15 mM (NH_4_)_2_SO_4_, 600 μM MgSO_4_·7H_2_O, 60 μM CaCl_2_·2H_2_O, 7.1 μM MnSO_4_·H_2_O] with 1% glucose and 22 μM Fe_2_SO_4_·7H_2_0 per liter). For high density cultures of *A. tumefaciens* (i.e., OD_600_ = 3 and OD_600_ = 4), the bacteria were cultured in LB broth for extended time periods (e.g., greater than 24 h), and tenfold dilutions of the culture were used to assess the optical density at 600 nm in the linear range of 0–1. For cloning, *Escherichia coli* strain DH5α was grown on tryptic soy agar (BD) or in Luria–Bertani (LB) broth. When appropriate, growth media were supplemented with kanamycin (45 μg mL^−1^ for *E. coli* and 300 μg mL^−1^ for *A. tumefaciens*), sucrose (5%), or IPTG (100 μM).

### Construction and complementation of *A. tumefaciens* deletion strains

#### Mutagenesis of A. tumefaciens vtlR, abcR1, abcR2, and vrsA

The *vtlR* gene (*atu2186*) was mutated utilizing an unmarked gene excision strategy previously described^22,26^. An approximately 1-kb fragment of the upstream region of *atu2186* was amplified via PCR with *A. tumefaciens* genomic DNA, primers *atu2186*-Up-For and *atu2186*-Up-Rev, and *Taq* polymerase (Monserate Biotechnology Group). Similarly, an approximately 1-kb fragment of the downstream region of *atu2186* was amplified with primers *atu2186*-Dn-For and *atu2186*-Dn-Rev. All oligonucleotides are listed in Supplementary Table S7. These fragments were then digested with the appropriate restriction enzymes, and subsequently phosphorylated with polynucleotide kinase (Monserate Biotechnology Group). Fragments were then combined in a single ligation with digested pNPTS138 and T4 DNA ligase (Monserate Biotechnology Group)^45^. The resulting plasmid was introduced into *A. tumefaciens* C58 by electroporation. Following electroporation of this plasmid into C58 primary integration of this non-replicating plasmid was confirmed using primer pairs USP003/*atu2186*-con-For and USP003/*atu2186*-con-Rev. Counter-selection on 5% sucrose resulted in excision of the integrated plasmid. Deletion of the *atu2186* (*vtlR*) locus was confirmed using primer pair *atu2186*-con-For/*atu2186*-con-Rev. All plasmid constructs are listed in Supplementary Table S8. This method was utilized to construct the *abcR1*, *abcR2*, and *abcR1/2* deletion constructs and strains.

#### In-trans complementation of A. tumefaciens str. C58:: ∆vtlR and A. tumefaciens str. C58:: ∆vrsA

Complementation of the *A. tumefaciens vtlR* deletion strain was done using IPTG-inducible overexpression plasmid pSRK-Km^33^. Briefly, the *A. tumefaciens vtlR* gene was amplified via PCR with *A. tumefaciens* genomic DNA, primers *atu2186*-comp-For and *atu2186*-comp-Rev and *Taq* polymerase. All oligonucleotides are listed in Supplementary Table S1. The fragment was digested with the appropriate restriction enzymes, and subsequently ligated into digested pSRK-Km. The resulting plasmid was then introduced into the *A. tumefaciens vtlR* strain by electroporation. The strain harboring the complementation plasmid was selected for on AT-agar plates supplemented with kanamycin (45 μg mL^−1^). All plasmid constructs are listed in Supplementary Table S2.

#### In-trans heterologous complementation of A. tumefaciens vtlR

Heterologous complementation was carried out as described above. For complementation with *lsrB* from *Sinorhizobium meliloti*, the *lsrB* gene (*SMc01226*) was amplified via PCR with *S. meliloti* 1021 genomic DNA, primers *lsrB*-comp-For and *lsrB*-comp-Rev and *Taq* polymerase. All oligonucleotides are listed in Supplementary Table S7. The fragment was digested with the appropriate restriction enzymes, and ligated into digested pSRK-Km. The resulting plasmid was then introduced into the *A. tumefaciens vtlR* deletion strain by electroporation. All plasmid constructs are listed in Supplementary Table S8.

For complementation with *vtlR* from *Brucella abortus*, the *vtlR* gene (*bab1_1517*) was amplified via PCR with *B. abortus* 2308 genomic DNA, primers *vtlR*-comp-For and *vtlR*-comp-Rev and *Taq* polymerase. All oligonucleotides are listed in Supplementary Table S7. The fragment was digested with the appropriate restriction enzymes, and ligated into digested pSRK-Km. The resulting plasmid was then introduced into the *A. tumefaciens vtlR* strain by electroporation. All plasmid constructs are listed in Supplementary Table S8.

#### Site-directed mutagenesis of VtlR binding motif in vrsA promoter

Phusion High-Fidelity DNA polymerase (New England BioLabs) was used to amplify a 1 kbp fragment from 554 nt upstream to 179 bp downstream of the *vrsA* coding sequence, using purified *A. tumefaciens* str. C58 genomic DNA primers USP204 and USP205. The 1 kbp amplicon was gel purified using the E.Z.N.A. gel extraction kit (Omega Bio-tek), A-tailed with *Taq* DNA polymerase (New England BioLabs), and ligated into vector pGEM-T Easy (Promega) to generate plasmid pJEH158. Plasmid pJEH158 was transformed into *E. coli* strain DH10B for propagation. Amplicon sequence was confirmed using Sanger sequencing (Genewiz).

For site-directed mutagenesis, 10 ng of plasmid pJEH158 served as template with 125 ng of each primer, USP206 and USP207. Phusion High-Fidelity DNA polymerase was used with the following cycling parameters. Initial denaturation: 95 °C, 30 s. Cyclic amplification: 95 °C, 30 s/55 °C, 1 min/68 °C, 4 min, for a total of 18 cycles in a volume of 50 μL. Following column purification of reaction products with E.Z.N.A. cycle pure kit (Omega Bio-tek) to 40 μL, 1 μL of *DpnI* and was added and the mixture incubated for 2 h at 37 °C. 5 μL of this reaction was then used to transform chemically competent *E. coli* strain DH10B. From a pool of transformants, multiple candidate mutagenized plasmids were purified and subjected to Sanger sequencing. Multiple mutant plasmids were identified and one, pJEH159, was retained for further use.

The entire 1-kb fragment originally cloned into pGEM-T Easy was sub-cloned from pJEH159 into the suicide plasmid pNPTS138 using engineered restriction sites for SpeI and SphI. The product of this sub-cloning, pJEH060, was verified by Sanger sequencing and transformed into the conjugative mating strain of *E. coli*, S17-1 λ*pir*.

Allelic replacement of the 1-kb region encompassing *vrsA* and its mutated promoter was performed using the same steps as for generating the ∆*vrsA* mutant strain of *A. tumefaciens* C58, generating strain C58-JEH169. A 2,027 bp amplicon from this strain generated using Phusion High-Fidelity DNA polymerase and primers USP152 and USP153 was used for sequence confirmation.

### Potato tumor assay

To test the virulence of the constructs, tumor formation on disks of red potato was measured. Organic, red potatoes were scrubbed to remove dirt and debris, sterilized in dilute bleach for 20 min, and finally sterilized with UV light for no less than 20 min. Potato disks were created by coring the potatoes and cutting the cores into 0.5-cm-wide slices. The slices were placed onto an agar plate with no added nutrients, and each plate had five technical replicates. The disks were inoculated with the indicated strains, which were grown overnight in ATGN. The overnight cultures were diluted to an optical density (600 nm) of 0.06 prior to inoculation, then ten microliters of the strain was placed on each potato disk. The plates were sealed with parafilm and left undisturbed at room temperature for 4 weeks. The tumor formation was counted at day 14 and 21.

### Static biofilm assay

To test the ability of a strain to form a biofilm, a static biofilm assay was performed. Overnight cultures of each strain were grown in LB. The following morning each culture was subcultured to an optical density (600 nm) of 0.1, and once the cultures reach exponential growth, they were diluted to an optical density (600 nm) of 0.05. Three milliliters of each culture was placed in a nine well polystyrene plate. Previously, polyvinyl chloride coverslips had been placed in each well, and then the plates were UV sterilized for no less than 20 min. After inoculation, the plates were incubated at room temperature for 48 h. The coverslips were rinsed to remove excess or weakly attached organisms, then stained with 0.1% crystal violet. After staining, the excess crystal violet was rinsed off and the adherent crystal violet was re-solubilized in 33% acetic acid. Three hundred microliters of re-solubilized crystal violet and culture were loaded into a 96 well plate. The absorbance of the crystal violet solution was measured at 600 nm (A_600nm_), and the optical density of the culture at 600 nm was measured (OD_600nm_). For data presentation biofilm formation is normalized to growth using the formula A_600nm_/OD_600nm_ and expressed relative to biofilm formation by the wild-type background.

### Northern blot analysis

*Agrobacterium tumefaciens* RNA was isolated from cultures using the methodology previously described^22,26^. Ten micrograms of RNA were separated on a denaturing 10% polyacrylamide gel with 7 M urea and 1 × TBE (89 mM Tris base, 89 mM boric acid, 2 mM EDTA). To determine size, a low molecular weight DNA ladder (New England Biolabs) was labelled with [γ-^32^P]ATP (PerkinElmer) and polynucleotide kinase (Monserate Biotechnology Group). Following electrophoresis, the ladder and RNA samples were transferred to a Amersham Hybond™-N^+^ membrane (GE Heathcare) by electroblotting in 1 × TBE buffer. Samples were then UV cross-linked to the membrane, and membranes were then pre-hybridized in ULTRAhyb^®^-Oligo Buffer (Ambion) for 1 h at 45 °C in a rotating hybridization oven. Oligonucleotide probes were end-labelled with [γ-^32^P]ATP and polynucleotide kinase. All oligonucleotides are listed in Supplementary Table S7. Radiolabeled probes were incubated with pre-hybridized membranes at 45 °C in a rotating hybridization oven overnight. The following day, membranes were washed four times with 2 × SSC (300 mM sodium chloride and 30 mM sodium citrate), 1 × SSC, 0.5 × SSC, and 0.25 × SSC at 45 °C in a rotating hybridization oven for 30 min each. Each SSC washing solution contained 0.1% sodium dodecyl sulphate (SDS). Membranes were exposed to X-ray film and visualized by autoradiography.

### Protein purification

Recombinant *A. tumefaciens* VtlR (rVtlR) was constructed utilizing the *Strep*-tag II system (IBA), and subsequently cloned and expressed in *E. coli* BL21 cells. The coding region of *atu2186* was amplified via PCR using *A. tumefaciens* C58 genomic DNA as a template, primers rAtu2186-For and rAtu2186-Rev and *Taq* polymerase (Monserate Biotechnology Group). All oligonucleotides are listed in Supplementary Table S7. The DNA was then digested with BsaI and ligated into pASK-IBA7, which encodes an amino-terminal Strep-tag II on the protein of interest. Following sequencing of the plasmid, the *E. coli* BL21 strain harboring rVtlR-pIBA7 was grown to an OD_600_ nm of 0.7 before recombinant gene expression was induced by 200 μg mL^−1^ anhydrotetracycline (AHT). Following 3 h of constant shaking at 37 °C, cultures were collected by centrifugation (4,200×*g* for 10 min at 4 °C) and lysed with CelLytic B (Sigma) in the presence of the protease inhibitor phenylmethanesulfonylfluoride (PMSF). The supernatant from the lysed cells was cleared by centrifugation (14,000×*g* for 10 min at 4 °C) and passed through a Strep-Tactin Sepharose affinity column. The column was then washed two times with Buffer W (100 mM Tris–HCl, 300 mM NaCl, pH 8.0) and the rVtlR was eluted with 2.5 mM desthiobiotin in Buffer W. The degree of purity of the rVtlR was high as judged by visualization of a single band on SDS-PAGE.

### Electrophoretic mobility shift assays (EMSAs)

EMSAs with rVtlR were carried out as previously described^22^. All EMSAs were done in a final volume of 20 μL reaction mixture that included a binding buffer composed of 10 mM Tris–HCl (pH 7.4), 50 mM KCl, 1 mM dithiothreitol, 6% glycerol, 50 μg mL^−1^ bovine serum albumin and 50 μg mL^−1^ salmon sperm DNA. DNA fragments of the *abcR1*, *abcR2*, *atu1667,* and *vrsA* promoter regions were amplified by PCR using *A. tumefaciens* C58 genomic DNA at a template, gene-specific primers and *Taq* polymerase (Monserate Biotechnology Group). All oligonucleotides are listed in Supplementary Table S7. Fragments were then run on a 0.8% agarose gel, purified and end-labelled with [γ-^32^P]ATP (PerkinElmer) and polynucleotide kinase (Monserate Biotechnology Group). Increasing amounts of rVtlR were added to DNA fragments in binding buffer, and subsequently incubated at room temperature for 30 min. In some gels, non-radiolabeled specific DNA (i.e. promoters of *abcR1* and *vrsA*) or non-radiolabeled non-specific DNA (i.e. promoter of *abcR2*) were added to reactions in 50 × molar concentrations. Binding reactions were run on 6% native polyacrylamide gels in 0.5 × TBE running buffer for 1 h. Gels were dried onto 3 mm Whatman paper using a vacuum gel drier system and visualized by autoradiography.

### RNA-sequencing

#### RNA extraction and precipitation

RNA extractions were carried out as previously described^22,24,26^. *Agrobacterium* strains were grown in triplicate to an OD_600_ nm of 1.0 with constant shaking at 28 °C. An equal amount of 1:1 ethanol-acetone was added to cultures and stored at − 80 °C. For RNA isolation, the cell/ethanol-acetone mixtures were thawed and pelleted at 16,000×*g* for 3 min. RNA was isolated from cells by use of TRIzol reagents (Invitrogen) followed by ethanol precipitation. Following RNA isolation, genomic DNA was removed with DNase I (2 U; Thermo Fisher Scientific), where 30 μg of RNA was incubated with DNase I for 1 h at 37 °C. Samples were then cleaned up by phenol–chloroform extractions and subsequent ethanol precipitation. RNA samples were resuspended in nuclease-free H_2_O and purity of each sample was checked with a NanoDrop 1,000 spectrophotometer (Thermo Fisher Scientific). All samples had an *A*_*260*_*/A*_*280*_ ratio of ~ 2.0 and a concentration yield of ~ 1 μg μL^−1^. RNA samples (10 μg total) were then submitted to the Bioinformatics Institute at Virginia Tech for RNA-seq analysis.

#### Stranded RNA library construction for prokaryotic RNA-Seq

The RNA-sequencing libraries were prepared as described previously by our group^43^. Briefly, 1 μg of total RNA with RIN ≥ 8.0 was depleted of rRNA using Illumina's Ribo-Zero rRNA Removal Kit (Gram-Positive and Gram-Negative Bacteria) (P/N MRZB12424, Illumina, CA, USA). The depleted RNA is fragmented and converted to first strand cDNA using reverse transcriptase and random primers using Illumina’s TruSeq Stranded mRNA HT Sample Prep Kit (Illumina, RS-122-2103). This is followed by second strand synthesis using polymerase I and RNAse H, and dNTPs that contain dUTP instead of dTTP. The cDNA fragments then go through end repair, addition of a single ‘A’ base, and then ligation of adapters and indexed individually. The products are then purified and the second strand digested with N-Glycosylase, thus resulting in stranded template. The template molecules with the adapters are enriched by 10 cycles of PCR to create the final cDNA library. The library generated is validated using Agilent 2100 Bioanalyzer and quantitated using Quant-iT dsDNA HS Kit (Invitrogen) and qPCR. 16 individually indexed cDNA libraries were pooled and sequenced on Illumina NextSeq to get a minimum of 25 million reads.

#### Illumina NextSeq sequencing

The Illumina NextSeq sequencing was carried out as described previously by our group^43^. Briefly, the libraries are clustered and sequenced using, NextSeq 500/550 High Output kit V2 (150 cycles) (P/N FC-404-2002) to 2 × 75 cycles to generate ~ 50 million paired end reads. The Illumina NextSeq Control Software v2.1.0.32 with Real Time Analysis RTA v2.4.11.0 was used to provide the management and execution of the NextSeq 500 and to generate BCL files. The BCL files were converted to FASTQ files and demultiplexed using bcl2fastq Conversion Software v2.20.

#### RNA-Seq data processing and analysis

The *Agrobacterium tumefaciens* (strain C58) gene and genome sequences, as well as corresponding annotations from NCBI (https://www.ncbi.nlm.nih.gov/) were used as a reference. Raw reads were quality-controlled and filtered with FastqMcf^46^, resulting in an average of 1,936 Mbp (1,703–2,329 Mbp) nucleotides. The remaining reads were mapped to the gene reference using BWA with default parameters^47^. Quality control of the sequence data is depicted in Supplementary Fig. S6. Differential expression of genes was calculated using the edgeR package in R software (https://www.r-project.org/), with Benjamini–Hochberg adjusted P values of 0.05 considered to be significant^48^.

### Quantitative reverse transcriptase PCR (qRT-PCR)

Total RNA isolated from *A. tumefaciens* str. C58 and *A. tumefaciens* ∆*vtlR* for the performance of RNA-seq analysis above was utilized to perform confirmatory qRT-PCR as previously stated^49^. Moreover, qRT-PCR was also carried out to compare gene expression in *A. tumefaciens* C58 and *A. tumefaciens vrsA*-SD. cDNA was generated from the final RNA preparation using a SuperScript III cDNA synthesis system (Invitrogen, Carlsbad, CA, USA) according to the manufacturer's protocol, and this cDNA was used for real-time PCR employing a SYBR green PCR supermix (Roche, Mannheim, Germany). For these experiments, primers for 16S rRNA were used as a control, while gene-specific primers were used for evaluating relative levels of *atu5161*, *avhB5*, *avhB11*, *atu5118*, *atu3253*, and *atu3368* mRNAs (Supplementary Table S7). Parameters for PCR included a single denaturing step for 5 min at 95 °C, followed by 40 cycles (denature for 15 s at 95 °C, anneal for 15 s at 51 °C, and extend for 15 s at 72 °C) of amplification. Fluorescence from SYBR green incorporation into double-stranded DNA was measured with an iCycler machine (Bio-Rad), and the relative abundance of mRNA was determined using the Pfaffl equation^50^.

## Supplementary information

Supplementary file1

## References

[1] Maddocks SE, Oyston PC (2008). Structure and function of the LysR-type transcriptional regulator (LTTR) family proteins. Microbiology.

[2] Henikoff S, Haughn GW, Calvo JM, Wallace JC (1988). A large family of bacterial activator proteins. Proc. Natl. Acad. Sci. USA.

[3] Kovacikova G, Lin W, Skorupski K (2010). The LysR-type virulence activator AphB regulates the expression of genes in *Vibrio cholerae* in response to low pH and anaerobiosis. J. Bacteriol..

[4] Liu Z (2011). *Vibrio cholerae* anaerobic induction of virulence gene expression is controlled by thiol-based switches of virulence regulator AphB. Proc. Natl. Acad. Sci. USA.

[5] Liu Z (2016). Differential thiol-based switches jump-start *Vibrio cholerae* pathogenesis. Cell Rep.

[6] Privett BR (2017). Identification of a small molecule activator for AphB, a LysR-type virulence transcriptional regulator in *Vibrio cholerae*. Biochemistry.

[7] Taylor JL (2012). The crystal structure of AphB, a virulence gene activator from *Vibrio cholerae*, reveals residues that influence its response to oxygen and pH. Mol. Microbiol..

[8] Craven SH (2009). Inducer responses of BenM, a LysR-type transcriptional regulator from *Acinetobacter baylyi* ADP1. Mol. Microbiol..

[9] Ezezika OC, Collier-Hyams LS, Dale HA, Burk AC, Neidle EL (2006). CatM regulation of the *benABCDE* operon: Functional divergence of two LysR-type paralogs in *Acinetobacter baylyi* ADP1. Appl. Environ. Microbiol..

[10] Ruangprasert A, Craven SH, Neidle EL, Momany C (2010). Full-length structures of BenM and two variants reveal different oligomerization schemes for LysR-type transcriptional regulators. J. Mol. Biol..

[11] Dangel AW, Gibson JL, Janssen AP, Tabita FR (2005). Residues that influence in vivo and in vitro CbbR function in *Rhodobacter sphaeroides* and identification of a specific region critical for co-inducer recognition. Mol. Microbiol..

[12] Smith SA, Tabita FR (2002). Up-regulated expression of the cbb(I) and cbb(II) operons during photoheterotrophic growth of a ribulose 1,5-bisphosphate carboxylase-oxygenase deletion mutant of *Rhodobacter sphaeroides*. J. Bacteriol..

[13] Tichi MA, Tabita FR (2002). Metabolic signals that lead to control of CBB gene expression in *Rhodobacter capsulatus*. J. Bacteriol..

[14] Akakura R, Winans SC (2002). Constitutive mutations of the OccR regulatory protein affect DNA bending in response to metabolites released from plant tumors. J. Biol. Chem..

[15] Wang L, Helmann JD, Winans SC, The A (1992). *tumefaciens* transcriptional activator OccR causes a bend at a target promoter, which is partially relaxed by a plant tumor metabolite. Cell.

[16] Kullik I, Toledano MB, Tartaglia LA, Storz G (1995). Mutational analysis of the redox-sensitive transcriptional regulator OxyR: Regions important for oxidation and transcriptional activation. J. Bacteriol..

[17] Zheng M, Aslund F, Storz G (1998). Activation of the OxyR transcription factor by reversible disulfide bond formation. Science.

[18] Luo L (2005). Two new *Sinorhizobium meliloti* LysR-type transcriptional regulators required for nodulation. J. Bacteriol..

[19] Lu D, Tang G, Wang D, Luo L (2013). The *Sinorhizobium meliloti* LysR family transcriptional factor LsrB is involved in regulation of glutathione biosynthesis. Acta Biochim. Biophys. Sin. (Shanghai).

[20] Tang G, Wang Y, Luo L (2014). Transcriptional regulator LsrB of *Sinorhizobium meliloti* positively regulates the expression of genes involved in lipopolysaccharide biosynthesis. Appl. Environ. Microbiol..

[21] Tang G (2017). Regulation of cysteine residues in LsrB proteins from *Sinorhizobium meliloti* under free-living and symbiotic oxidative stress. Environ. Microbiol..

[22] Sheehan LM, Budnick JA, Blanchard C, Dunman PM, Caswell CC (2015). A LysR-family transcriptional regulator required for virulence in *Brucella abortus* is highly conserved among the alpha-proteobacteria. Mol. Microbiol..

[23] Becker A, Overlöper A, Schlüter J-P, Reinkensmeier J (2014). Riboregulation in plant-associated α-proteobacteria. RNA Biol..

[24] Caswell CC (2012). Identification of two small regulatory RNAs linked to virulence in *Brucella abortus* 2308. Mol. Microbiol..

[25] Overloper A (2014). Two separate modules of the conserved regulatory RNA AbcR1 address multiple target mRNAs in and outside of the translation initiation region. RNA Biol..

[26] Sheehan LM, Caswell CC (2017). A 6-nucleotide regulatory motif within the AbcR small RNAs of *Brucella abortus* mediates host–pathogen interactions. MBio.

[27] Torres-Quesada O (2013). Independent activity of the homologous small regulatory RNAs AbcR1 and AbcR2 in the legume symbiont *Sinorhizobium meliloti*. PLoS One.

[28] Torres-Quesada O (2014). Genome-wide profiling of Hfq-binding RNAs uncovers extensive post-transcriptional rewiring of major stress response and symbiotic regulons in *Sinorhizobium meliloti*. RNA Biol..

[29] Wilms I, Voss B, Hess WR, Leichert LI, Narberhaus F (2011). Small RNA-mediated control of the *Agrobacterium tumefaciens* GABA binding protein. Mol. Microbiol..

[30] Storz G, Vogel J, Wassarman KM (2011). Regulation by small RNAs in bacteria: Expanding frontiers. Mol. Cell.

[31] Sheehan LM, Caswell CC (2018). An account of evolutionary specialization: The AbcR small RNAs in the *Rhizobiales*. Mol. Microbiol..

[32] Tang G (2018). The LsrB protein is required for *Agrobacterium tumefaciens* interaction with host plants. Mol. Plant Microbe Interact..

[33] Khan SR, Gaines J, Roop RM, Farrand SK (2008). Broad-host-range expression vectors with tightly regulated promoters and their use to examine the influence of TraR and TraM expression on Ti plasmid quorum sensing. Appl. Environ. Microbiol..

[34] Munch R (2005). Virtual Footprint and PRODORIC: An integrative framework for regulon prediction in prokaryotes. Bioinformatics.

[35] Wilms I, Overloper A, Nowrousian M, Sharma CM, Narberhaus F (2012). Deep sequencing uncovers numerous small RNAs on all four replicons of the plant pathogen *Agrobacterium tumefaciens*. RNA Biol..

[36] Kemner JM, Liang X, Nester EW (1997). The *Agrobacterium tumefaciens* virulence gene *chvE* is part of a putative ABC-type sugar transport operon. J. Bacteriol..

[37] Matthysse AG, Jaeckel P, Jeter C (2008). *attG* and *attC* mutations of *Agrobacterium tumefaciens* are dominant negative mutations that block attachment and virulence. Can J Microbiol.

[38] Chen L, Chen Y, Wood DW, Nester EW (2002). A new type IV secretion system promotes conjugal transfer in *Agrobacterium tumefaciens*. J. Bacteriol..

[39] Kim J, Heindl JE, Fuqua C (2013). Coordination of division and development influences complex multicellular behavior in *Agrobacterium tumefaciens*. PLoS One.

[40] Heindl JE (2015). Discrete responses to limitation for iron and manganese in *Agrobacterium tumefaciens*: Influence on attachment and biofilm formation. J. Bacteriol..

[41] Eiamphungporn W, Nakjarung K, Prapagdee B, Vattanaviboon P, Mongkolsuk S (2003). Oxidant-inducible resistance to hydrogen peroxide killing in *Agrobacterium tumefaciens* requires the global peroxide sensor-regulator OxyR and KatA. FEMS Microbiol. Lett..

[42] Nakjarung K, Mongkolsuk S, Vattanaviboon P (2003). The *oxyR* from *Agrobacterium tumefaciens*: Evaluation of its role in the regulation of catalase and peroxide responses. Biochem. Biophys. Res. Commun..

[43] Budnick JA, Sheehan LM, Kang L, Michalak P, Caswell CC (2018). Characterization of three small proteins in *Brucella abortus* linked to fucose utilization. J. Bacteriol..

[44] Liang Y, Aoyama T, Oka A (1998). Structural characterization of the *virB* operon on the hairy-root-inducing plasmid A4. DNA Res..

[45] Spratt BG, Hedge PJ, te Heesen S, Edelman A, Broome-Smith JK (1986). Kanamycin-resistant vectors that are analogues of plasmids pUC8, pUC9, pEMBL8 and pEMBL9. Gene.

[46] Aronesty E (2013). Comparison of sequencing utility programs. Open Bioinform. J..

[47] Li H, Durbin R (2009). Fast and accurate short read alignment with Burrows–Wheeler transform. Bioinformatics.

[48] Robinson MD, McCarthy DJ, Smyth GK (2010). edgeR: A Bioconductor package for differential expression analysis of digital gene expression data. Bioinformatics.

[49] Sheehan LM, Budnick JA, Roop RM, Caswell CC (2015). Coordinated zinc homeostasis is essential for the wild-type virulence of *Brucella abortus*. J. Bacteriol..

[50] Pfaffl MW (2001). A new mathematical model for relative quantification in real-time RT-PCR. Nucleic Acids Res..

