# Supplementary material for: A central role for the transcriptional regulator VtlR in small RNA-mediated gene regulation in Agrobacterium tumefaciens
Source: Sci Rep. 2020 Sep 11;10:14968. doi: 10.1038/s41598-020-72117-0 (PMC7486931; doi:10.1038/s41598-020-72117-0)
Supplement: Supplementary file 1 — Supplementary file1 [file 41598_2020_72117_MOESM1_ESM.docx]

**Supplementary Tables, Figures, and Blot Images**

**A central role for the transcriptional regulator VtlR in small RNA-mediated gene regulation in *Agrobacterium tumefaciens***

James A. Budnick^1,*^, Lauren M. Sheehan^1,*^, Miranda. J. Ginder^2^, Kevin C. Failor^2^, Julia. M. Perkowski^2^, John. F. Pinto^2^, Kirsten A. Kohl^1^, Lin Kang^3^, Pawel Michalak^1,3,4^, Li Luo^5^, Jason E. Heindl^2,a^, and Clayton C. Caswell^1,a^

^1^Center for One Health Research, Virginia-Maryland College of Veterinary Medicine, Virginia Tech, Blacksburg, VA, 24060, USA.

^2^Department of Biological Sciences, University of the Sciences in Philadelphia, Philadelphia, PA, 19104, USA.

^3^Edward Via College of Osteopathic Medicine, Blacksburg, VA, 24060, USA.

^4^Institute of Evolution, Haifa University, Haifa, 3498838, Israel.

^5^Shanghai Key Laboratory of Bio-energy Crops, School of Life Sciences, Plant Science Center, Shanghai University, Shanghai, 200444, China.

*J.A.B and L.M.S contributed equally to this work.

^a^Address correspondence to Clayton Caswell, [caswellc@vt.edu](mailto:caswellc@vt.edu) or Jason Heindl, [j.heindl@usciences.edu](mailto:j.heindl@usciences.edu).





**Figure S1: *S. meliloti* LsrB does not control expression of the AbcR sRNAs.**

Northern blot analysis was carried out with *S. meliloti* wild-type (1021) and a deletion of *lsrB* analyzing the expression of the AbcR sRNAs.

**
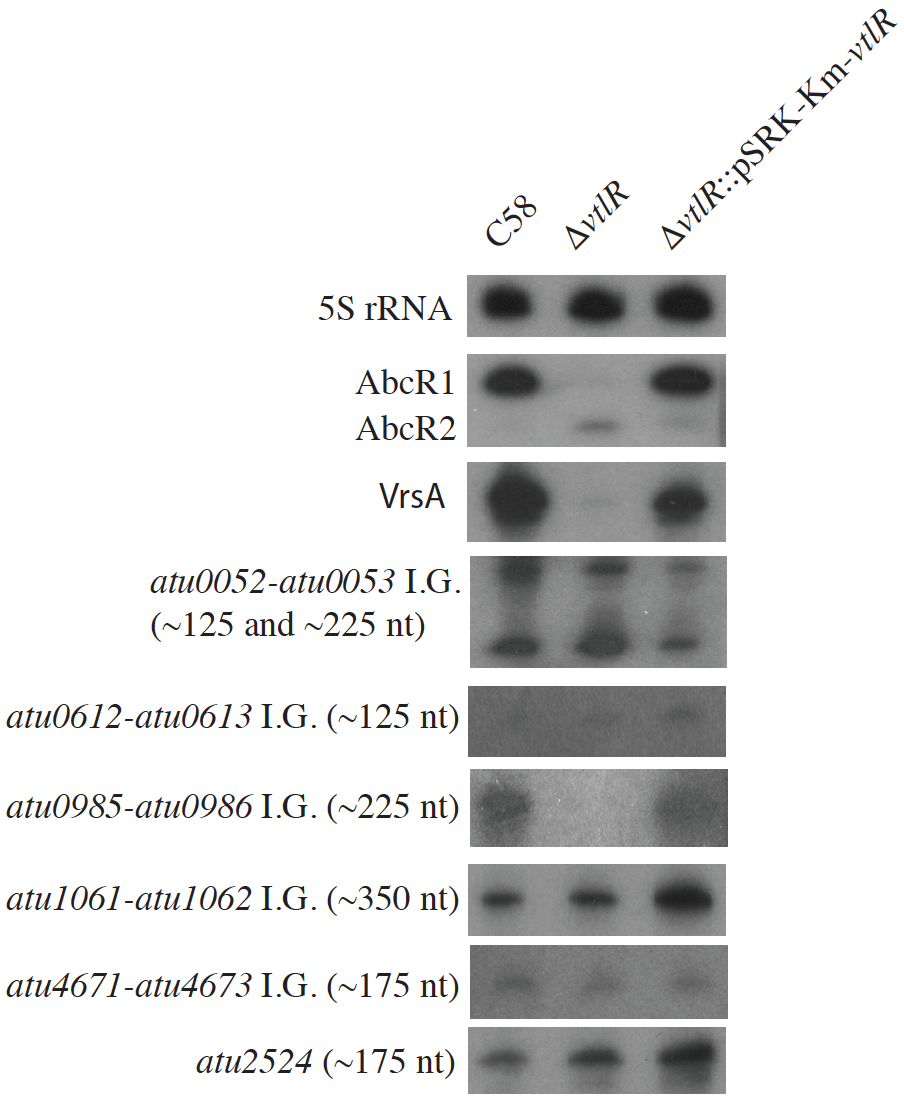
**

**Figure S2: Expression of novel sRNAs in *A. tumefaciens* str. C58, Δ*vtlR*, and Δ*vtlR*-pSRK-Km-*vtlR.***

Northern blot analysis to measure expression of AbcR1, AbcR2, VrsA, Atu1667, and novel trans- and cis-encoded sRNAs identified to be putatively differentially expressed in Table 3.2 in *A. tumefaciens* str. C58, **Δ***vtlR*, and **Δ***vtlR*-pSRK-Km-*vtlR.*

**Figure S3: Characterization of *A. tumefaciens* Δ*vrsA*.**

A. In vitro growth kinetics of *A. tumefaciens* strains. The *A. tumefaciens* str. C58 and **Δ***vrsA* strains were grown in LB broth, and at specified time points, samples from each culture were taken, serial diluted and plated on AT-agar plates to determine colony forming units (CFUs). Data represents average CFUs per ml ± the standard deviation of results from triplicate samples.

B. Tumor formation of *A. tumefaciens* str. C58 and Δ*vrsA* in experimentally infected potatoes. Sterile potato discs were inoculated with C58 or Δ*vrsA*, and the number of tumors was counted 14- and 21-days post infection.

C. Biofilm formation by *A. tumefaciens* strains. Data are means and standard deviations from three separate experiments normalized to C58.

D. Swim ring diameters were measured after single-colony inoculation into low density swim agar and incubation at room temperature. The data are the mean of nine independent experiments.


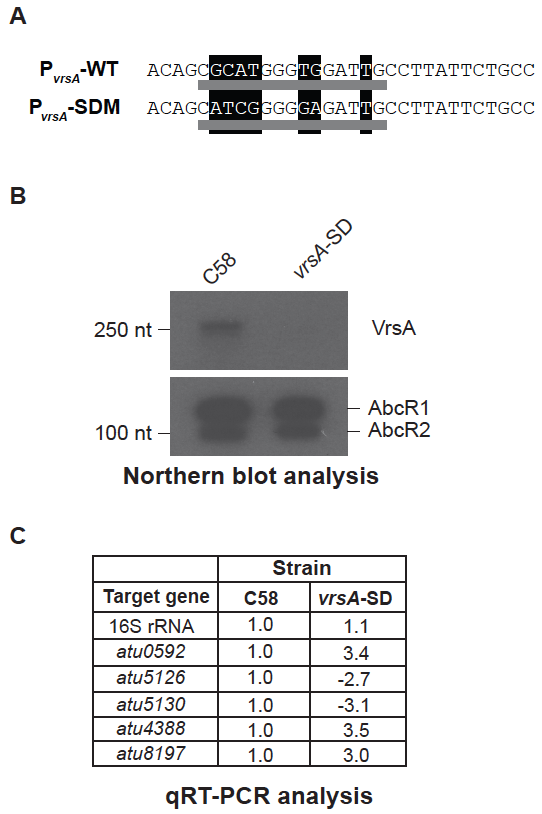


**Figure S4: Mutation of the VtlR binding site in the *vrsA* promoter leads to gene expression differences similar to the Δ*vrsA* strain*.***

A. Schematic of *vrsA* promoter. The VtlR binding site is underlined with a gray bar, and the nucleotide substitutions are denoted.

B. Northern blot analyses for levels of VrsA and AbcR sRNAs in *Agrobacterium* strains. RNA was isolated from wild-type *A. tumefaciens* (C58) and the A. tumefaciens strain carrying a mutated *vrsA* promoter (*vrsA*-SD). 10 μg of RNA per lane was separated in urea-denaturing polyacrylamide gels, and following transfer to a positively charged membrane, probes for VrsA or the AbcR sRNAs were used to visualize sRNA levels.

C. qRT-PCR analysis of gene expression in *Agrobacterium* strains. RNA was isolated from C58 or *vrsA*-SD, reverse transcribed, and the resulting cDNA was assessed by qPCR for specific target genes. The numbers represent the fold difference in levels of the indicated target gene mRNA, and 16S rRNA was used as a control.


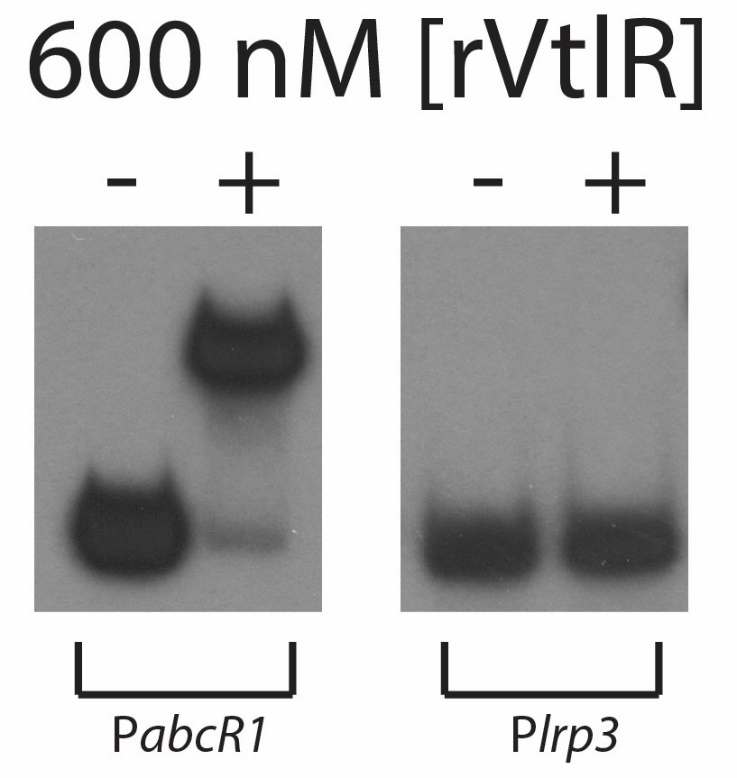


**Figure S5: *A. tumefaciens* VtlR does not bind to the promoter region of *lrp3.***

Since it was previously shown how LsrB bound to the promoter region of *lrp3* in *S. meliloti*, we sought to test the ability of VtlR to bind *lrp3* in *A. tumefaciens*. Electrophoretic mobility shift assays (EMSAs) were carried out with recombinantly purified VtlR (rVtlR) and ^32^P-radiolabeled *lrp3* promoter region (P*_lrp3_*). A (+) represents the addition of 600 nM rVtlR, where a (–) represents no protein added to the binding reaction mixture. The promoter region of *abcR1* was used as a control for rVtlR activity (P*_abcR1_*).

**Figure S6:** **Sequencing summary after quality control.**

Bars in different colors represent nucleotides from all (Total, grey) sequencing quality greater than or equal to 20 (Q20, yellow), and sequencing quality greater than or equal to 30 (Q30, blue). Samples S31-S33: 3 replicates of *A. tumefaciens* str. C58 cultured in LB broth; S34-S36: 3 replicates of *A. tumefaciens* str. C58::Δ*abcR1* cultured in LB broth; S37-39: 3 replicates of *A. tumefaciens* str. C58::Δ*abcR2* cultured in LB broth; S40-42: 3 replicates of *A. tumefaciens* str. C58::Δ*vtlR* cultured in LB broth.

Table S1: Differential gene expression in *A. tumefaciens* str. C58::Δ*abcR1*

| **Gene Designation** | **Description** | **Log_2_ fold change (Δ*abcR1* vs. C58) in gene expression** |
| --- | --- | --- |
|  | **Transport Systems and Membrane Proteins** |  |
| Atu0126 | membrane lipoprotein | 1.5 |
| Atu0157 | ABC transporter, substrate binding protein | 2.8 |
| Atu0158 | ABC transporter, membrane spanning protein | 2.7 |
| Atu0159 | ABC transporter, nucleotide binding/ATPase protein | 2.2 |
| Atu1201 | ABC transporter, nucleotide binding/ATPase protein | 1.8 |
| Atu1202 | ABC transporter, substrate binding protein | 2.3 |
| Atu1879 | ABC transporter, substrate binding protein (amino acid) | 2.2 |
| Atu2143 | ABC transporter, membrane spanning protein (amino acid) | 1.8 |
| Atu2287 | outer membrane heme receptor | -1.6 |
| Atu2348 | *chvE*, sugar binding protein | -1.5 |
| Atu2391 | ABC transporter, substrate binding protein (nitrate/sulfonate/taurine/bicarbonate) | 2.8 |
| Atu2422 | ABC transporter, substrate binding protein (amino acid) | 2.4 |
| Atu3041 | dipeptide ABC transporter substrate-binding protein | 1.7 |
| Atu3047 | oligopeptide ABC transporter permease | 1.9 |
| Atu3048 | oligopeptide ABC transporter permease | 1.9 |
| Atu3049 | oligopeptide ABC transporter substrate-binding protein | 1.6 |
| Atu3053 | peptide ABC transporter ATPase | 2.4 |
| Atu3063 | ABC transporter permease | 1.7 |
| Atu3269 | dipeptide ABC transporter substrate-binding protein | 2.2 |
| Atu3338 | *thuE*, ABC transporter, substrate binding protein (trehalose/maltose) | -1.6 |
| Atu3339 | *thuF*, ABC transporter, membrane spanning protein | -1.7 |
| Atu3340 | *thuG*, trehalose/maltose ABC transporter permease | -1.7 |
| Atu3341 | *thuK*, trehalose/maltose ABC transporter ATPase | -1.7 |
| Atu3342 | *thuA*, trehalose utilization-like protein | -1.7 |
| Atu3409 | oligopeptide ABC transporter substrate-binding protein (atu2409) | 1.7 |
| Atu3800 | *proX*, proline/glycine betaine ABC transporter substrate-ninding protein | 2.2 |
| Atu3801 | *proW*, proline/glycine betaine ABC transporter permease | 1.7 |
| Atu3802 | *proV*, proline/glycine betaine ABC transporter ATPase | 1.7 |
| Atu3821 | *rbsB*, ribose ABC transporter substrate-binding protein | -1.8 |
| Atu4031 | sugar ABC transporter permease | -1.6 |
| Atu4046 | glycine betaine ABC transporter substrate-binding protein | 1.5 |
| Atu4233 | amino acid ABC transporter substrate-binding protein | 2.1 |
| Atu4234 | amino acid ABC transporter permease | 2.1 |
| Atu4235 | amino acid ABC transporter ATPase/permease | 1.5 |
| Atu4284 | amino acid ABC transporter substrate-binding protein | 1.5 |
| Atu4370 | sugar ABC transporter ATPase | -1.9 |
| Atu4371 | sugar ABC transporter permease | -1.8 |
| Atu4423 | ABC transporter permease | -2.1 |
| Atu4577 | ABC transporter substrate binding protein | 3.5 |
| Atu4578 | ABC transporter permease | 2.8 |
| Atu4579 | ABC transporter permease | 1.9 |
| Atu4678 | amino acid ABC transporter substrate-binding protein | 1.6 |
| Atu4695 | oligopeptide ABC transporter substrate-binding protein | 3.7 |
| Atu4696 | oligopeptide ABC transporter permease | 3.4 |
| Atu4697 | oligopeptide ABC transporter permease | 2.6 |
| Atu4700 | oligopeptide ABC transporter ATPase | 2.3 |
| Atu4701 | oligopeptide ABC transporter ATPase | 2.3 |
| Atu4723 | ABC transporter permease | 1.6 |
| Atu4755 | amino acid ABC transporter substrate-binding protein | 2.2 |
| Atu5006 | *socA*, Deoxyfructosyl-amino Acid Transporter Periplasmic Binding Protein | 1.7 |
| Atu5531 | ABC transporter substrate binding protein | 1.9 |
| Atu6026 | *nocQ*, ABC transporter, membrane spanning protein nopaline | 1.6 |
| Atu6027 | *nocT*, ABC transporter, substrate binding protein (nopaline) | 2.2 |
|  |  |  |
|  | **Transcription and Translation** |  |
| Atu4718 | GntR family transcriptional regulator | 1.5 |
|  |  |  |
|  | **Chemotaxis and Motility** |  |
| Atu3725 | methyl-accepting chemotaxis protein | 2.5 |
|  |  |  |
|  | **Metabolism, Signaling, and Enzymatic Processes** |  |
| Atu0666 | *glcE*, glycolate oxidase subunit | -2.1 |
| Atu0667 | *glcF*, glycolate oxidase iron-sulfur subunit | -1.8 |
| Atu0946 | dehydrogenase | 2.4 |
| Atu2127 | *caiB*, L-carnitine dehydratase | 2.1 |
| Atu2128 | *iivG*, acetolactate synthase 2 catalytic subunit | 1.5 |
| Atu2386 | *dht*, dihydropyrimidinase | 1.7 |
| Atu2497 | hydrolase | 2.1 |
| Atu2499 | Isochorismatase | 3.0 |
| Atu2500 | *rutA*, monooxygenase (pyrimidine utilization protein A) | 3.2 |
| Atu3046 | oxidoreductase | 1.7 |
| Atu4153 | aldehyde dehydrogenase | 2.4 |
| Atu4236 | dehydrogenase | 1.8 |
| Atu4377 | oxidoreductase | -3.1 |
| Atu4379 | *cy2*, cytochrome c2 | -1.8 |
| Atu4418 | *gcdH*, glutaryl-CoA dehydrogenase | 2.1 |
| Atu4698 | Catalyzes the deamination of cytosine to uracil and ammonia | 2.2 |
| Atu4699 | *trzA*, N-ethylammeline chlorohydrolase | 2.4 |
|  |  |  |
|  | **Phage Related Genes** |  |
| Atu0954 | *gp34*, phage head portal protein | -1.7 |
| Atu0957 | *gp36*, phage phi-C31 major capsid gp36-like protein | -2.0 |
|  |  |  |
|  | **Hypothetical Proteins** |  |
| Atu0058 | hypothetical protein | -2.7 |
| Atu0945 | hypothetical protein | 1.5 |
| Atu0958 | hypothetical protein | -1.7 |
| Atu0961 | hypothetical protein | -1.5 |
| Atu0964 | hypothetical protein | -1.7 |
| Atu0965 | hypothetical protein | -1.7 |
| Atu0967 | hypothetical protein | -1.9 |
| Atu2146 | hypothetical protein | 1.7 |
| Atu2469 | hypothetical protein | -1.7 |
| Atu2471 | hypothetical protein | -1.6 |
| Atu2498 | hypothetical protein | 2.1 |
| Atu2541 | hypothetical protein | -2.7 |
| Atu3942 | hypothetical protein | -2.7 |
| Atu4374 | hypothetical protein | -1.8 |
| Atu4375 | hypothetical protein | -2.1 |
| Atu4376 | hypothetical protein | -2.9 |
| Atu4378 | hypothetical protein | -2.8 |
| Atu4484 | hypothetical protein | -1.5 |
| Atu4580 | hypothetical protein | 1.9 |
| Atu8141 | hypothetical protein | -1.6 |
| Atu8177 | hypothetical protein | 1.6 |

**Table 1. Summarized Δ*abcR1* RNA-seq dataset**

RNA-seq analysis was performed using total cellular RNA from *Agrobacterium* strains grown in LB medium to late exponential phase, and genes with expression ≥3-fold dysregulated are shown. Cells highlighted in grey represent genes also found in Table S3 (Δ*vtlR* RNA-seq dataset). Dataset sorted based on gene designation.

Table S2: Differential gene expression in *A. tumefaciens* str. C58::Δ*abcR2*

| **Gene Designation** | **Description** | **Log_2_ fold change (Δ*abcR2* vs. C58) in gene expression** |
| --- | --- | --- |
| Atu2109 | *rnpB*, RNAase P RNA | -1.9 |
| Atu2478 | hypothetical protein | 1.5 |
| Atu3667 | *panD*, aspartate alpha-decarboxylase | 1.7 |
| Atu3676 | putative siderophore biosynthesis protein | 1.6 |
| Atu3752 | hypothetical protein | 2.0 |
| Atu4088 | transcriptional regulator | 1.6 |
| Atu4376 | hypothetical protein | -1.5 |
| Atu5115 | hypothetical protein | 1.5 |

**Table 2. Summarized Δ*abcR2* RNA-seq dataset**

RNA-seq analysis was performed using total cellular RNA from *Agrobacterium* strains grown in LB medium to late exponential phase, and genes with expression ≥3-fold dysregulated are shown.

Table S3: Differential gene expression in *A. tumefaciens* str. C58::Δ*vtlR*

| **Gene Designation** | **Description** | **Log_2_ fold change (Δ*vtlR* vs. C58) in gene expression** |
| --- | --- | --- |
|  | **Transport Systems and Membrane Proteins** |  |
| Atu0063 | *frcB,* ABC transporter, substrate binding protein (sugar) | 1.7 |
| Atu0157 | ABC transporter, substrate binding protein | 1.9 |
| Atu0158 | ABC transporter, membrane spanning protein | 1.6 |
| Atu0159 | ABC transporter, nucleotide binding/ATPase protein | 1.7 |
| Atu0394 | ABC transporter, substrate binding protein (sugar) | -1.7 |
| Atu0593 | *aglG*, ABC transporter, membrane spanning protein | -2.1 |
| Atu0843 | P type cation (metal) transporter, ATPase component | -1.6 |
| Atu0893 | ABC transporter, nucleotide binding/ATPase protein | -1.7 |
| Atu0894 | ABC transporter, membrane spanning protein | -2.1 |
| Atu1398 | ABC transporter, membrane spanning protein (amino acid) | 1.9 |
| Atu1399 | ABC transporter, substrate binding protein (amino acid) | 2.6 |
| Atu1403 | ABC transporter, membrane spanning protein | 1.9 |
| Atu1579 | ABC transporter, membrane spanning protein (amino acid) | -1.7 |
| Atu1877 | OmpA family protein | -1.5 |
| Atu2287 | outer membrane heme receptor | -1.5 |
| Atu2346 | *gguB*, ABC transporter, membrane spanning protein (sugar) | -3.1 |
| Atu2347 | *gguA*, ABC transporter, nucleotide binding/ATPase protein (sugar) | -2.3 |
| Atu2348 | *chvE*, sugar binding protein | -2.5 |
| Atu2391 | ABC transporter, substrate binding protein (nitrate/sulfonate/taurine/bicarbonate) | 2.1 |
| Atu2422 | ABC transporter, substrate binding protein (amino acid) | 1.5 |
| Atu2492 | *mtbA*, permease (Atu2492) | 2.1 |
| Atu2505 | ABC transporter, substrate binding protein (sugar) | 2.4 |
| Atu2708 | MFS permease | -3.7 |
| Atu3102 | sugar ABC transporter substrate-binding protein | 1.8 |
| Atu3113 | sugar ABC transporter permease | 1.7 |
| Atu3114 | sugar ABC transporter substrate-binding protein | 1.7 |
| Atu3151 | sugar ABC transporter substrate-binding protein | 1.5 |
| Atu3165 | sorbitol/mannitol ABC transporter substrate-binding protein | 1.8 |
| Atu3185 | *ugpB*, ABC transporter, substrate binding protein (glycerol-3-phosphate) | 2.9 |
| Atu3187 | *ugpE*, ABC-type sugar transport system, permease component | 1.7 |
| Atu3198 | ribose ABC transporter substrate-binding protein | 2.2 |
| Atu3200 | ribose ABC transporter ATPase | 1.9 |
| Atu3202 | RND multidrug efflux transporter | -3.1 |
| Atu3203 | RND multidrug efflux membrane permease | -2.9 |
| Atu3222 | ribose ABC transporter substrate-binding protein | 2.8 |
| Atu3236 | sugar ABC transporter ATPase | 1.7 |
| Atu3237 | sugar ABC transporter permease | 1.6 |
| Atu3238 | sugar ABC transporter permease | 1.7 |
| Atu3239 | sugar ABC transporter substrate-binding protein | 2.7 |
| Atu3253 | ABC transporter substrate binding protein | 4.5 |
| Atu3254 | dicarboxylate ABC transporter ATPase | 3.3 |
| Atu3255 | dicarboxylate ABC transporter permease | 3.2 |
| Atu3298 | *dctA*, C4-dicarboxylate transporter (Atu3298) | 1.8 |
| Atu3338 | *thuE*, ABC transporter, substrate binding protein (trehalose/maltose) | 1.9 |
| Atu3352 | sugar ABC transporter substrate-binding protein | 2.2 |
| Atu3368 | periplasmic mannitol-binding protein | 3.7 |
| Atu3370 | sugar ABC transporter permease | 1.5 |
| Atu3371 | sugar ABC transporter ATPase | 2.9 |
| Atu3372 | sugar ABC transporter substrate-binding protein | 3.6 |
| Atu3455 | oligopeptide ABC transporter substrate-binding protein | 1.9 |
| Atu3533 | sugar ABC transporter substrate-binding protein | 2.0 |
| Atu3576 | *xylF*, ABC transporter, substrate binding protein (xylose) | 3.3 |
| Atu3804 | permease | -1.7 |
| Atu3881 | sugar ABC transporter substrate-binding protein | 1.8 |
| Atu3893 | sugarl ABC transporter permease | 2.2 |
| Atu3894 | sugar ABC transporter permease | 2.0 |
| Atu3895 | sugar ABC transporter ATPase | 2.7 |
| Atu3896 | sugar ABC transporter ATPase | 3.2 |
| Atu4017 | *kgtP*, MFS permease | -2.8 |
| Atu4032 | sugar ABC transporter ATPase | 1.8 |
| Atu4033 | sugar ABC transporter substrate-binding protein | 2.8 |
| Atu4123 | branched chain amino acid ABC transporter substrate-binding protein | 2.4 |
| Atu4124 | ABC transporter ATP-binding protein | 2.1 |
| Atu4192 | oligopeptide ABC transporter substrate-binding protein | 2.4 |
| Atu4423 | ABC transporter permease | -1.8 |
| Atu4468 | *sitD*, ABC transporter, membrane spanning protein (iron transport) | -1.7 |
| Atu4469 | *sitC*, ABC transporter, membrane spanning protein (iron) | -1.6 |
| Atu4471 | *sitA*, ABC transporter, substrate binding protein (iron | -1.6 |
| Atu4534 | amino acid ABC transporter substrate-binding protein | 2.1 |
| Atu4577 | ABC transporter substrate binding protein | 2.0 |
| Atu4578 | ABC transporter permease | 2.0 |
| Atu4626 | oligopeptide ABC transporter substrate-binding protein | 1.5 |
| Atu4661 | *agpA*, ABC transporter, substrate binding protein (alpha-galactoside) | 2.0 |
| Atu4710 | MFS permease | 2.5 |
| Atu4784 | *afuA*, ABC-type Fe3+ transport system, periplasmic component | 2.1 |
| Atu4842 | sugar ABC transporter substrate-binding protein | 2.0 |
| Atu4843 | sugar ABC transporter ATPase | 2.1 |
| Atu5005 | *socB*, Deoxyfructosyl-amino acid ABC-type Membrane Transporter | 1.9 |
| Atu5006 | *socA*, Deoxyfructosyl-amino Acid Transporter Periplasmic Binding Protein | 3.2 |
| Atu5126 | *attA1*, ABC transporter, nucleotide binding/ATPase protein(putrescine) | -2.7 |
| Atu5127 | *attA2*, ABC transporter, membrane spanning protein (mannopine) | -3.2 |
| Atu5128 | *attB*, ABC transporter, membrane spanning protein (mannopine) | -3.1 |
| Atu5129 | *attC*, ABC transporter, substrate binding protein (Atu5129) | -3.6 |
| Atu5130 | *attD*, attachment protein | -3.0 |
| Atu6027 | *nocT*, ABC transporter, substrate binding protein (nopaline) | 1.5 |
|  |  |  |
|  | **Transcription and Translation** |  |
| Atu0484 | two component response regulator | 2.7 |
| Atu0828 | *betI*, transcriptional regulator | 2.4 |
| Atu1296 | *divK*, two component response regulator | 1.5 |
| Atu2186 | *vtlR* | -6.2 |
| Atu2187 | transcriptional regulator, ArsR family | 1.7 |
| Atu2350 | *gbpR*, transcriptional regulator, LysR family | -1.6 |
| Atu2384 | transcriptional regulator, TetR family | 1.7 |
| Atu3252 | *exuR*, transcriptional regulator, GntR family | 1.6 |
| Atu4782 | *drrA*, two component response regulator | 1.7 |
| Atu5116 | *rctB*, transcriptional regulator protein | -2.7 |
| Atu5119 | two component response regulator | -3.4 |
| Atu5121 | two component response regulator | -2.8 |
|  |  |  |
|  | **Secretion Systems** |  |
| Atu5162 | *avhB1*, type IV secretion protein AvhB1 | -7.4 |
| Atu5163 | *avhB2*, type IV secretion protein AvhB2 | -6.3 |
| Atu5164 | *avhB3*, type IV secretion protein AvhB3 | -6.9 |
| Atu5165 | *avhB4*, type IV secretion protein AvhB4 | -7.6 |
| Atu5166 | *avhB5*, type IV secretion protein AvhB5 | -7.6 |
| Atu5167 | *avhB6*, type IV secretion protein AvhB6 | -6.7 |
| Atu5168 | *avhB7*, type IV secretion protein AvhB7 | -6.0 |
| Atu5169 | *avhB8*, type IV secretion protein AvhB8 | -6.3 |
| Atu5170 | *avhB9*, type IV secretion protein AvhB9 | -6.6 |
| Atu5171 | *avhB10*, type IV secretion protein AvhB10 | -6.1 |
| Atu5172 | *avhB11*, type IV secretion protein AvhB11 | -4.5 |
|  |  |  |
|  | **Metabolism, Signaling, and Enzymatic Processes** |  |
| Atu0462 | endolysin | -2.3 |
| Atu0594 | *aglA*, alpha-glucosidase | -1.9 |
| Atu0666 | *glcE*, glycolate oxidase subunit | -1.9 |
| Atu0667 | *glcF*, glycolate oxidase iron-sulfur subunit | -1.9 |
| Atu0704 | *dgoK*, 2-dehydro-3-deoxygalactonate kinase | -1.9 |
| Atu0811 | *mqo*, malate:quinone oxidoreductase | -3.6 |
| Atu0830 | *betA*, choline dehydrogenase | 2.0 |
| Atu0946 | dehydrogenase | 2.7 |
| Atu1277 | *nuoH*, NADH dehydrogenase subunit H | -1.9 |
| Atu1432 | *aceF*, branched-chain alpha-keto acid dehydrogenase subunit E2 | -1.6 |
| Atu1661 | *sthA*, soluble pyridine nucleotide transhydrogenase | -1.7 |
| Atu1956 | *rpoB*, DNA-directed RNA polymerase subunit beta | -1.5 |
| Atu2109 | *rpnB*, RNAase P RNA | -1.6 |
| Atu2127 | *caiB*, L-carnitine dehydratase | 2.9 |
| Atu2128 | *iivG*, acetolactate synthase 2 catalytic subunit | 1.5 |
| Atu2224 | *aldA*, aldehyde dehydrogenase | 3.8 |
| Atu2497 | hydrolase | 1.6 |
| Atu2499 | Isochorismatase | 1.9 |
| Atu2500 | *rutA*, monooxygenase (pyrimidine utilization protein A) | 2.3 |
| Atu2511 | *dat*, D-amino acid aminotransferase | -1.7 |
| Atu3137 | C4-dicarboxylate-binding protein | 2.5 |
| Atu3256 | zinc-binding dehydrogenase | 1.7 |
| Atu3354 | pyrroloquinoline-quinone-dependent quinate dehydrogenase | -2.9 |
| Atu3471 | *bkdB*, branched-chain alpha-keto acid dehydrogenase subunit E2 | 3.0 |
| Atu3472 | *bkdA2*, 2-oxoisovalerate dehydrogenase beta subunit | 3.8 |
| Atu3473 | *bkdA1*, 2-oxoisovalerate dehydrogenase alpha subunit | 3.8 |
| Atu3474 | *mmgC*, acyl-CoA dehydrogenase | 1.6 |
| Atu3475 | acetyl-CoA acetyltransferase | 3.0 |
| Atu3478 | *mccB*, 3-methylcrotonoyl-CoA carboxylase beta subunit | 2.5 |
| Atu3479 | *mccA*, 3-methylcrotonyl-CoA carboxylase alpha subunit | 2.6 |
| Atu3480 | *hmgL*, hydroxymethylglutaryl-CoA lyase | 2.0 |
| Atu3667 | *panD*, aspartate alpha-decarboxylase | 2.2 |
| Atu3736 | *tktA*, transketolase | -1.7 |
| Atu4153 | aldehyde dehydrogenase | 2.6 |
| Atu4416 | phosphopantetheinyl transferase | 2.1 |
| Atu4418 | *gcdH*, glutaryl-CoA dehydrogenase | 3.4 |
| Atu4709 | *fdsD*, NAD-dependent formate dehydrogenase delta subunit | 2.0 |
| Atu4740 | zinc-binding dehydrogenase | -3.5 |
| Atu4825 | dehydrogenase | 1.7 |
| Atu5004 | *socC*, deoxyfructose oxidoreductase | 1.9 |
| Atu5123 | *atrC*, acetolactate synthase catalytic subunit | -5.8 |
| Atu5124 | *atrB*, glutamate-1-semialdehyde aminotransferase | -5.9 |
| Atu5173 | dehydrogenase | -2.1 |
| Atu6000 | *torf6*, agrocinopine synthase | 1.7 |
| Atu6007 | *mas1*, Mannopine synthase | 1.5 |
| Atu6010 | *tms2*, indole acetimide hydrolase | 1.9 |
| Atu6012 | *ipt*, isopentenyl transferase | 1.5 |
| Atu6013 | 6a protein, Cytokinin glycosidase | 1.6 |
| Atu6179 | *virC2*, virA/G regulated protein | 1.6 |
|  |  |  |
|  | **Phage Related Genes** |  |
| Atu0954 | *gp34*, phage head portal protein | -1.7 |
| Atu0956 | *gp35*, phage prohead protease | -1.7 |
| Atu0957 | *gp36*, phage phi-C31 major capsid **gp36**-like protein | -3.6 |
| Atu8126 | putative phage tail protein I | -2.3 |
|  |  |  |
|  | **Conjugation** |  |
| Atu5108 | *traG*, conjugal transfer protein | -4.0 |
| Atu5109 | *traD*, conjugal transfer protein | -4.3 |
| Atu5110 | *traC*, conjugal transfer protein | -5.2 |
|  |  |  |
|  | **Polysaccharide Biosynthesis** |  |
| Atu3326 | *exoF*, exopolysaccharide production protein | -1.6 |
| Atu3327 | *exoY*, succinoglycan exopolysaccharide synthesis protein | -1.8 |
| Atu4049 | *exoP*, exopolysaccharide polymerization/transport protein | -2.0 |
| Atu4050 | *exoN*, UTP-glucose-1-phosphate uridylyltransferase | -2.0 |
| Atu4054 | *exoL*, succinoglycan biosynthesis protein | -1.5 |
| Atu4059 | *exoV*, succinoglycan biosynthesis protein | -2.1 |
|  |  |  |
|  | **Hypothetical Proteins** |  |
| Atu0056 | hypothetical protein | 2.2 |
| Atu0058 | hypothetical protein | -2.3 |
| Atu0104 | hypothetical protein | 1.7 |
| Atu0115 | hypothetical protein | 1.7 |
| Atu0240 | hypothetical protein | 1.9 |
| Atu0250 | hypothetical protein | 1.5 |
| Atu0277 | hypothetical protein | -1.7 |
| Atu0452 | hypothetical protein | -1.6 |
| Atu0453 | hypothetical protein | -2.3 |
| Atu0454 | hypothetical protein | -2.1 |
| Atu0463 | hypothetical protein | -2.1 |
| Atu0468 | hypothetical protein | 2.3 |
| Atu0604 | hypothetical protein | 1.6 |
| Atu0772 | hypothetical protein | 1.6 |
| Atu0824 | hypothetical protein | 1.6 |
| Atu0853 | hypothetical protein | 1.9 |
| Atu0945 | hypothetical protein | 2.4 |
| Atu0958 | hypothetical protein | -3.0 |
| Atu0961 | hypothetical protein | -2.1 |
| Atu0964 | hypothetical protein | -2.7 |
| Atu0965 | hypothetical protein | -2.4 |
| Atu0967 | hypothetical protein | -2.9 |
| Atu1009 | hypothetical protein | 1.7 |
| Atu1031 | hypothetical protein | -2.9 |
| Atu1427 | hypothetical protein | 1.7 |
| Atu1469 | hypothetical protein | 1.5 |
| Atu1587 | hypothetical protein | 2.1 |
| Atu1634 | hypothetical protein | 2.4 |
| Atu1667 | hypothetical protein | -4.3 |
| Atu1716 | hypothetical protein | 1.8 |
| Atu1727 | hypothetical protein | 1.5 |
| Atu1766 | hypothetical protein | 2.8 |
| Atu1773 | hypothetical protein | -1.9 |
| Atu2146 | hypothetical protein | 2.4 |
| Atu2248 | hypothetical protein | 1.7 |
| Atu2345 | *gguC*, hypothetical protein | -2.5 |
| Atu2433 | hypothetical protein | 1.7 |
| Atu2469 | hypothetical protein | 1.9 |
| Atu2478 | hypothetical protein | 2.9 |
| Atu2498 | hypothetical protein | 1.8 |
| Atu2524 | hypothetical protein | 1.8 |
| Atu2541 | hypothetical protein | -2.3 |
| Atu2543 | hypothetical protein | 2.2 |
| Atu2768 | hypothetical protein | 1.5 |
| Atu3124 | hypothetical protein | 1.5 |
| Atu3189 | hypothetical protein | 2.6 |
| Atu3470 | hypothetical protein | 2.1 |
| Atu3638 | hypothetical protein | 1.5 |
| Atu3645 | hypothetical protein | 2.9 |
| Atu3752 | hypothetical protein | 3.1 |
| Atu3822 | hypothetical protein | 2.1 |
| Atu3841 | hypothetical protein | 1.5 |
| Atu3891 | hypothetical protein | 3.5 |
| Atu3892 | hypothetical protein | 2.0 |
| Atu3940 | hypothetical protein | 2.2 |
| Atu3942 | hypothetical protein | -2.3 |
| Atu4020 | hypothetical protein | -2.3 |
| Atu4179 | hypothetical protein | 1.8 |
| Atu4183 | hypothetical protein | 2.2 |
| Atu4185 | hypothetical protein | 2.5 |
| Atu4213 | hypothetical protein | 1.9 |
| Atu4442 | hypothetical protein | 2.3 |
| Atu4443 | hypothetical protein | 1.8 |
| Atu4484 | hypothetical protein | -1.7 |
| Atu4527 | hypothetical protein | 1.9 |
| Atu4670 | hypothetical protein | -2.1 |
| Atu4727 | hypothetical protein | 1.9 |
| Atu4780 | hypothetical protein | -1.8 |
| Atu4820 | hypothetical protein | 1.5 |
| Atu5008 | hypothetical protein | 1.6 |
| Atu5037 | hypothetical protein | 1.8 |
| Atu5117 | hypothetical protein (VBP1- VirD2-binding protein) -NTPase that might energize the recruitment of T-complex to the transport site | -4.5 |
| Atu5118 | hypothetical protein | -8.3 |
| Atu5161 | hypothetical protein | -6.9 |
| Atu6163 | hypothetical protein | 1.5 |
| Atu8021 | hypothetical protein | 1.9 |
| Atu8083 | hypothetical protein | -1.5 |
| Atu8120 | hypothetical protein | 1.9 |
| Atu8127 | hypothetical protein | -2.1 |
| Atu8141 | hypothetical protein | -1.9 |
| Atu8161 | hypothetical protein | -3.8 |
| Atu8166 | hypothetical protein | 1.5 |
| Atu8177 | hypothetical protein | 1.7 |
| Atu8178 | hypothetical protein | -1.6 |
| Atu8201 | hypothetical protein | 1.6 |

**Table 3. Summarized Δ*vtlR* RNA-seq dataset**

RNA-seq analysis was performed using total cellular RNA from *Agrobacterium* strains grown in LB medium to late exponential phase, and genes with expression ≥3-fold dysregulated are shown. Cells highlighted in grey represent genes also found in Table S1 (Δ*abcR1* RNA-seq dataset). Dataset sorted based on gene designation.

Table S4: qRT-PCR confirmation of gene dysregulation of several VtlR target genes.

|  | **Strain** | | |
| --- | --- | --- | --- |
| **Target gene** | **2308** | **Δ*vtlR*-RNAseq** | **Δ*vtlR*-qRT-PCR** |
| 16S rRNA | 1.0 | -0.5 | 1.0 |
| *atu5161* | 1.0 | -6.8 | -25.0 |
| *avhB5* | 1.0 | -7.6 | -2.7 |
| *avhB11* | 1.0 | -4.5 | -2.3 |
| *atu5118* | 1.0 | -8.3 | -30.3 |
| *atu3253* | 1.0 | 4.5 | 1.4 |
| *atu3368* | 1.0 | 3.6 | 5.1 |

Table S5: Differential gene expression of putative sRNAs identified in Δ*vtlR* from Wilms et al. [1]

| **trans-encoded sRNAs** |  |  |  |  |
| --- | --- | --- | --- | --- |
| **Replicon** | **flanking genes** | **Log_2_ fold change (Δ*vtlR* vs. C58) in gene expression** | **Confirmed via northern blot?** | **Differentially expressed in Δ*vtlR?*** |
| Circular Chromosome | *atu0052-atu0053* | 1.4 | Yes | No |
| Circular Chromosome | *atu0523-atu0524* | 1.6 | No | - |
| Circular Chromosome | *atu0612-atu0613* | 1.3 | Yes | No |
| Circular Chromosome | *atu0654-atu8135* | 1.7 | No | - |
| Circular Chromosome | *atu0923-atu0924* | 2.7 | No | - |
| Circular Chromosome | *atu0985-atu0986* | -2.5 | Yes | Yes |
| Circular Chromosome | *atu1061-atu1062* | 1.6 | Yes | No |
| Circular Chromosome | *atu2014-atu2015* | 1.4 | No | - |
| Circular Chromosome | *atu2108-atu2110* | -1.5 | No | - |
| Circular Chromosome | *atu2122-atu2123* | 3.3 | No | - |
| Circular Chromosome | *atu2555-atu2557* | 2.6 | No | - |
| Circular Chromosome | *atu2770-atu2771* | 1.3 | No | - |
| Linear Chromosome | *atu3126-atu3127* | 1.1 | No | - |
| Linear Chromosome | *atu3367-atu3368* | 2.8 | No | - |
| Linear Chromosome | *atu4670-atu4671* | -2.8 | No | - |
| Linear Chromosome | *atu4671-atu4673* | 1.1 | Yes | No |
| Linear Chromosome | *atu4727-atu4728* | 2.1 | No | - |

| **cis-encoded sRNAs** |  |  |  |  |
| --- | --- | --- | --- | --- |
| **Replicon** | **cis-encoded gene** | **Log_2_ fold change (Δ*vtlR* vs. C58) in gene expression** | **Confirmed via northern blot?** | **Differentially expressed in Δ*vtlR?*** |
| Circular Chromosome | *atu0468* | 2.3 | No | - |
| Circular Chromosome | *atu0604* | 1.6 | No | - |
| Circular Chromosome | *atu1727* | 1.5 | No | - |
| Circular Chromosome | *atu2478* | 2.9 | No | - |
| Circular Chromosome | *atu2524* | 1.8 | Yes | No |
| Linear Chromosome | *atu4670* | -2.1 | Yes | Yes |
| At plasmid | *atu5117* | -4.5 | No | No |

Table 6: Differential gene expression in *A. tumefaciens* str. C58::Δ*vrsA*

| **Gene Designation** | **Description** | **Log_2_ fold change (Δ*vrsA* vs. C58) in gene expression** |
| --- | --- | --- |
|  | **Transport Systems and Membrane Proteins** |  |
| Atu5130 | *attD,* attachment protein | -1.5 |
| Atu5129 | *attC,* ABC transporter substrate binding protein (mannopine) | -1.5 |
| Atu5126 | *attA1,* ABC transporter nucleotide binding/ATPase(putrescine) | -1.5 |
| Atu5127 | *attA2,* ABC transporter membrane spanning protein (mannopine) | -1.4 |
| Atu5128 | *attB,* ABC transporter membrane spanning protein (mannopine) | -1.3 |
| Atu0593 | *aglG,* ABC transporter, membrane spanning protein | 2.0 |
| Atu0592 | *aglF,* ABC transporter, membrane spanning protein | 2.1 |
| Atu0591 | *aglE,* ABC transporter, substrate binding protein | 2.3 |
|  |  |  |
|  | **Denitrification** |  |
| Atu4385 | hypothetical protein | 1.6 |
| Atu4391 | *norE,* nitric oxide reductase | 1.6 |
| Atu4382 | *nirK,* nitrite reductase | 1.8 |
| Atu4383 | hypothetical protein | 2.0 |
| Atu8197 | *norF,* NorF protein | 2.0 |
| Atu4386 | *norD,* nitric oxide reductase | 2.1 |
| Atu4387 | *norQ,* nitric oxide reductase | 2.1 |
| Atu4388 | *norB,* nitric oxide reductase, cytochrome b subunit | 2.2 |
| Atu4389 | *norC,* nitric oxide reductase, cytochrome c-containing subunit | 2.2 |
|  |  |  |
|  | **Hypothetical Proteins** |  |
| Atu4670 | hypothetical protein | -4.7 |

**Table 6. Summarized Δ*vrsA* RNA-seq dataset**

RNA-seq analysis was performed using total cellular RNA from *Agrobacterium* strains grown in LB medium to late exponential phase, and genes with expression ≥3-fold dysregulated are shown. Cells highlighted in grey represent genes also found in Table S3 (Δ*vtlR* RNA-seq dataset). Dataset sorted based on gene designation.

Table S7: Oligonucleotide primers used in this study.

| **Primer name** | **Sequence (5'->3')** |
| --- | --- |
| *atu2186*-Up-For | TAGGATCCTATTTCCATGTCTGCCCGCCAC |
| *atu2186*-Up-Rev | TGCCATGCCTATCCGGCCTTTC |
| *atu2186*-Dn-For | TTCTGACGTAAAAATAATCGACGTCG |
| *atu2186*-Dn-Rev | TAACTAGTTTTCTGGTCGGTTGGCGTAAT |
| *atu2186*-con-For | AACTGTTCAAGGGCAAGGTG |
| *atu2186*-con-Rev | AAAACCTCCAGAGGGGAACAGC |
| USP003 | CGCCAGGGTTTTCCCAGTCACGAC |
| *abcR1*-Up-For | TAGGATCCAGTCGGCCATCAGCCGCCAGGTCAGC |
| *abcR1*-Up-Rev | TCAACTGAGAGCTATTTGGTGTTTTT |
| *abcR1*-Dn-For | TTTTTACCCGCAAGAATTTTCTCCA |
| *abcR1*-Dn-Rev | TAACTAGTATGCCAGATCTGCGCAACGATCTTGC |
| *abcR2*-Up-For | TAGGATCCACGACGGTTGGTCTCGGCCAGGGCTG |
| *abcR2*-Up-Rev | TGACAGCTAACTAAAATATGGGAGTG |
| *abcR2*-Dn-For | TTTTTTGGAGCCGCCTGCCCG |
| *abcR2*-Dn-Rev | TAACTAGTATATCGTCGATGGTGAGCGCGCGG |
| *abcR*-con-For | TTCCGCGACTTCATCGTCGCCAAGGC |
| *abcR*-con-Rev | ACGTCTCTGTGAGCATCGTGGCAAGC |
| AbcR-Northern | AAAACCTCCAGAGGGGAACAGCTG |
| VrsA-Northern-Rev | GTGTTACGTTCATGACATGACCCTTTCTGA |
| Atu1667-Northern | CGAGTTCGGTTACGGTCTGACGATACTTGC |
| comp-*atu2186*-For | TACATATGATGGCAATGCCATTGGACTGGGA |
| comp-*atu2186*-Rev | TAGCTAGCTCAGAAGTTCCAGTTCCGCGCCTT |
| comp-*BavtlR*-For | TAGAGCTCGTGGTCGCACCGCTTGACTGGGAT |
| comp-*BavtlR*-Rev | TAGCTAGCTCAATAGGTCCAATTGCGGGCCTT |
| comp-*SmlsrB*-For | TACATATGATGGGGGATTCTATGTCGCTGGA |
| comp-*SmlsrB*-For | TAGCTAGCTCAGAAGTTCCAGTTTCTCGCTT |
| *abcR1*-At-EMSA-For | GCGGAACTGGAACTTCTGACGTAA |
| *abcR1*-At-EMSA-Rev | GAACTGGGAGGAAAAGCCACCGTG |
| *abcR2*-At-EMSA-For | CGGCGGCCCTCTTTTTTTACC |
| *abcR2*-At-EMSA-Rev | GCCGTGGGCAAGCGTGACAGCT |
| *atu5161*-EMSA-For | CATCTTTCACTTAAATTGCTTGC |
| *atu5161*-EMSA-Rev | CTATGATCATTCGAAAGAAAAGG |
| *avhB1*-EMSA-For | GCAAAGGGTAAACGGGCGAT |
| *avhB1*-EMSA-Rev | CGGCGTCCGACGCAAGGAATG |
| *chvE*-EMSA-For | GACAGTTATATGGATTCAGA |
| *chvE*-EMSA-Rev | GTCCTGTGCGAAAGCCGGCGC |
| *atrA*-EMSA-For | GCTCGGAGCGGCTTTCAAGA |
| *atrA*-EMSA-Rev | CTGGCCTTGGGCGCCGGTAT |
| *atrB*-EMSA-For | GATATCAGGGAGGGTTACCGC |
| *atrB*-EMSA-Rev | CCGGTCGTTGGGACCTGTTAGC |
| *atu0828*-EMSA-For | TTCAAGCCGCCTCGCGTGCTT |
| *atu0828*-EMSA-Rev | GAAATAATGATGCGCAAGC |
| *atu0484*-EMSA-For | ATCGTTGCGCGTTGTGGCT |
| *atu0484*-EMSA-Rev | AAAGACCGCCTGTCCACAAG |
| *atu2384*-EMSA-For | CATGCCGGCCGCCTCGG |
| *atu2384*-EMSA-Rev | TGACCGGCTGGTAAACATT |
| *atu2350*-EMSA-For | ATAGAATATATCGGTTTCCATA |
| *atu2350*-EMSA-Rev | CGAGGCTCTCTGACAAAGCGGTG |
| *atu2187*-EMSA-For | CTTCAAACTTGTCAGGCCG |
| *atu2187*-EMSA-Rev | TGCGACAGGCCGCTGATCT |
| *atu1296*-EMSA-For | TTTTTGTCTCGCGCAGCATTTCT |
| *atu1296*-EMSA-Rev | GATGAGGTCCGGGCGGTGCT |
| *atu5119*-EMSA-For | CGGCTGGGCAACTTTGTC |
| *atu5119*-EMSA-Rev | ATAATACGCTCTGACTTCAAG |
| *atu5116*-EMSA-For | GATGACTCTCCCGTCGTCAA |
| *atu5116*-EMSA-Rev | CGTCGGATCGACGTTTTCGT |
| *atu4782*-EMSA-For | GATTTGCTCGTGTCCCAGTGTC |
| *atu4782*-EMSA-Rev | CAATCCATTGAATGGTTACCGA |
| *atu3252*-EMSA-For | CCGACCGGCTGAGCTGCCCTC |
| *atu3252*-EMSA-Rev | GAACGGTAGGGCGGGAAAC |
| *atu5121*-EMSA-For | GGTGTGGCTGACTATTCGC |
| *atu5121*-EMSA-Rev | GGAGAGGCCGAAGAAGTGGC |
| *atu5118*-EMSA-For | GCAGCCGGCGTAAGCGCCGTTC |
| *atu5118*-EMSA-Rev | GTCGGTCGACATTTCTCAGGTCC |
| *vrsA*-EMSA-For | CATTTACTTTTTTAAAGATATGA |
| *vrsA*-EMSA-Rev | GAGATTTTGTAAGTCCCATGG |
| *atu2708*-EMSA-For | GCTGCAACGCTTTGCGATCGAT |
| *atu2708*-EMSA-Rev | AATAACCGATGAAAAACAGCGA |
| *atu0055*-EMSA-For | AACGCGATTGGTGTGGTTATGTGC |
| *atu0055*-EMSA-Rev | GACGTTTCGGGCCTAAACCCAA |
| *atu0157*-EMSA-For | ATGCGCCTGGCTGTGTTATCCT |
| *atu0157*-EMSA-Rev | AGCAGACGAAACAAGCGTCGC |
| *atu0463*-EMSA-For | CCAAAACAGTTCAATGCCGAAGAA |
| *atu0463*-EMSA-Rev | TGAGCCTTGCGATTTGCTTGTGC |
| *atu0323*-EMSA-For | GCGTCTTGCCCAGCGCGTTAA |
| *atu0323*-EMSA-Rev | TTTCTGCAAAAACTCAGATCGG |
| *atu3939*-EMSA-For | GGCTGTAGCTCAGCTGGGAGAG |
| *atu3939*-EMSA-Rev | CTAAACCCAAAACACCTGGA |
| *atu4669*-EMSA-For | TTCTGGCGCCTTCACCACCGC |
| *atu4669*-EMSA-Rev | CAGTTCACGGGCGCTCATGCG |
| rAtu2186-For | TAGGATCCATGGCAATGCCATTGGACTGGGATA |
| rAtu2186-Rev | TACTCGAGTCAGAAGTTCCAGTTCCGCGCCTTG |
| rLsrB-For | ATGGGGGATTCTATGTCGCTGGA |
| rLsrB-Rev | TCAGAAGTTCCAGTTTCTCGCTT |
| *atu0052-atu0053*-Rev | CAAATCTCTCCAGGTCCCGCAAGCTTCGCCGCCCA |
| *atu0654-atu8135*-For | GTCGGGGGCGGTGCCGCATCGTGCCGTTACGGAAG |
| *atu0654-atu8135*-Rev | CTTCCGTAACGGCACGATGCGGCACCGCCCCCGAC |
| *atu0923-atu0924*-For | GGAACTTTGCTGCATCGCCTTAGTTTTCAACGGGT |
| *atu0923-atu0924*-Rev | ACCCGTTGAAAACTAAGGCGATGCAGCAAAGTTCC |
| *atu2014-atu2015*-For | ATCGGCGAGGGTTGAGACCGTTGCCACCCCCCTCT |
| *atu2014-atu2015*-Rev | AGAGGGGGGTGGCAACGGTCTCAACCCTCGCCGAT |
| *atu2108-atu2110*-For | CCTTGCCACCACTGTCACCAGCGGCGCGGTGGGCTCTTA |
| *atu2108-atu2110*-Rev | TAAGAGCCCACCGCGCCGCTGGTGACAGTGGTGGCAAGG |
| *atu2122-atu2123*-For | GTCGGTTTGGCGTTTTTGTTCGTAGCGATGATGCT |
| *atu2122-atu2123*-Rev | AGCATCATCGCTACGAACAAAAACGCCAAACCGAC |
| *atu2770-atu2771*-For | GAACTCCTCGATGAGGACTGTTGGCAGCTTATCGT |
| *atu2770-atu2771*-Rev | ACGATAAGCTGCCAACAGTCCTCATCGAGGAGTTC |
| *atu3126-atu3127*-For | GGACCAATGCTTCAGGCTGATTGCGCAACCATGAC |
| *atu3126-atu3127*-Rev | GTCATGGTTGCGCAATCAGCCTGAAGCATTGGTCC |
| *atu3367-atu3368*-For | TGAAGCCGGACAGTCACTGACCAGCCTGTCCGGTT |
| *atu3367-atu3368*-Rev | AACCGGACAGGCTGGTCAGTGACTGTCCGGCTTCA |
| *atu4670-atu4671*-For | CGGTAATGCGGGTGATCCTACTCCTCCTCCCAAAG |
| *atu4670-atu4671*-Rev | CTTTGGGAGGAGGAGTAGGATCACCCGCATTACCG |
| *atu4671-atu4673*-Rev | CGAAAGATGCTCCACGGTGACGTTCTCCGA |
| *atu4727-atu4728*-Rev | AGGGTCTTGATGAATGATGATGTCATGCAACGCCT |
| *atu0523-atu0524*probeFor | CTTCTTGAATGGAATGATTCCATCCCGAGGTATAATCTCC |
| *atu0523-atu0524*probeRev | GGAGATTATACCTCGGGATGGAATCATTCCATTCAAGAAG |
| *atu0612-atu0613*probeRev | GAAAGGGGGGACAATCTCGCCACTTGGAAATTATCAATCC |
| *atu0985-atu0986*probeFor | TCAGCGCTCGCGGAAATCCGCAAAGACTGCCGAGGGACGC |
| *atu1061-atu1062*probeFor | GGCTTACGCTTCAGCATCAGGGCTGCGGCAGCAACGGCCT |
| *atu2555-atu2557*probeFor | TATCAACATCAACGCAATCCTTGGTCTTGTGTCCTGGAAT |
| *atu2555-atu2557*probeRev | ATTCCAGGACACAAGACCAAGGATTGCGTTGATGTTGATA |
| *atu4670-atu4671*-For | CGGTAATGCGGGTGATCCTACTCCTCCTCCCAAAG |
| *atu4670-atu4671*-Rev | CTTTGGGAGGAGGAGTAGGATCACCCGCATTACCG |
| *atu0468*-For-Northern | CATTTTCCCAAGACTGCTCCTCCGCTTCAATTGAT |
| *atu0468*-Rev-Northern | ATCAATTGAAGCGGAGGAGCAGTCTTGGGAAAATG |
| *atu0604*-For-Northern | TGGAGGCTCTTAACAGGGCATGGGTCGGTTGAG |
| *atu0604*-Rev-Northern | CTCAACCGACCCATGCCCTGTTAAGAGCCTCCA |
| *atu1727*-For-Northern | CCGGCATTTGAGACGCCGGGCCTGTGGAACTTATG |
| *atu1727*-Rev-Northern | CATAAGTTCCACAGGCCCGGCGTCTCAAATGCCGG |
| *atu2478*-For-Northern | GCCTTCAAGATCGCAGCAGTCATGTTGCCAATGAA |
| *atu2478*-Rev-Northern | TTCATTGGCAACATGACTGCTGCGATCTTGAAGGC |
| *atu2524*-Rev-Northern | CGCTACCGCGACTTCGCGGCAGCGTTTGATTTCGG |
| *atu5117*-For-Northern | GAACCATGCTCTCTCCCGATGGTGATCGCGAGGGA |
| *atu5117*-Rev-Northern | TCCCTCGCGATCACCATCGGGAGAGAGCATGGTTC |
| *atu4670*-Up-For | TAGGATCCGCAGACGTATTGCAAAAAGAAAC |
| *atu4670*-Up-Rev | TCCTGCCACAGGCAAAGGGC |
| *atu4670*-Dn-For | TCGTTGATGGTCTGACGATATTT |
| *atu4670*-Dn-Rev | TACTGCAGTGGCTTCGGAGACAAGCGAGG |
| 16S rRNA-RT-For | ATATTCGGAGGAACACCAGT |
| 16S rRNA-RT-Rev | ACCGAACAGTATACTGCCCG |
| *atu0592-*RT-For | TTATCGCTGGGCCTTCTTCG |
| *atu0592-*RT-Rev | GATTTGGCGATGTTGCCCCA |
| *atu5126-*RT-For | TTTCCTTCTGAACTTTCGGG |
| *atu5126-*RT-Rev | GATGCGCTTGATTTCGAACT |
| *atu5130-*RT-For | ATGCGCAGGCGGCTCTCTTT |
| *atu5130-*RT-Rev | CAGCGGACTGCACAATGACG |
| *atu8197-*RT-For | TTGCAAGAAGTTTCGCAGTG |
| *atu8197-*RT-Rev | AATCGTCGATGACACGCCTG |
| *atu4388-*RT-For | TCACCGAAGACAGCGTGTGC |
| *atu4388-*RT-Rev | TCACCATGGTCATCTTCACC |
| *atu5161-*RT-For | GCAGCGTCAGGGTCCATTTCAC |
| *atu5161-*RT-Rev | CCTCCAAAGCTTCTCGACGGG |
| *atu5118-*RT-For | GCAGGTCCGTACGTTGGCCCATA |
| *atu5118-*RT-Rev | CCGTATCGATCCTGCAAGTAGCG |
| *atu3253-*RT-For | TCGCCGCTGTCAGCTATTACGG |
| *atu3253-*RT-Rev | TCGGTGTCGTGTTGGCGC |
| *atu3368-*RT-For | TTGGCCGCGCCTGCCAT |
| *atu3368-*RT-Rev | CTGAATGGTGAAATTGCCGTCG |
| *avhB5-*RT-For | ATTCCGCCTCAAGGTTTCTGG |
| *avhB5-*RT-Rev | CGATGCGCTTGGTCCCG |
| *avhB11-*RT-For | GCCGTTTTCGACATTCCTTCAG |
| *avhB11-*RT-Rev | ACTGCCCGGGCAAGACGC |
| USP152 | GAGGGCTTTGTTCAGGAGTA |
| USP153 | CGTCGTTTACACTCCATTCACT |
| USP204 | ACTAGTGTTCTATCCCTATCGTCTCGGCA |
| USP205 | GCATGCAGATATGCGCTATATGCATGGC |
| USP206 | GGAACCATTGCACACAGCATCGGGGGAGATTGCCTTATTCTGCCTGTTC |
| USP207 | GAACAGGCAGAATAAGGCAATCTCCCCCGATGCTGTGTGCAATGGTTCC |
|  |  |

*Underlined sequences depict a restriction endonuclease recognition site.

Table S8: Plasmids used in this study.

| **Plasmid name** | **Description** | **Reference** |
| --- | --- | --- |
| pNPTS138 | Cloning vector; contains *sacB*; Kan^R^ | M.R.K. Alley, unpublished |
| pSRK-Km | pBBR1MCS-2-derived broad-host-range expression vector containing *lac* promoter and *lacI*^q^, *lacZ*α^+^, and Km^R^ | [2] |
| pASK-IBA7 | Recombinant protein expression vector; Amp^R^ | IBA |
| p*atu2186* | In-frame deletion of *atu2186* plus 1-kb of each flanking region in pNPTS138 | This study |
| p*abcR1* | In-frame deletion of *abcR1* plus 1-kb of each flanking region in pNPTS138 | This study |
| p*abcR2* | In-frame deletion of *abcR2* plus 1-kb of each flanking region in pNPTS138 | This study |
| P*atu4670* | In-frame deletion of *atu4670* plus 1-kb of each flanking region in pNPTS138 | This study |
| pSRK-Km-*AtvtlR* | Coding region of *atu2186* in pSRK-Km for complementation | This study |
| pSRK-Km-*BavtlR* | Coding region of *bab1_1517* in pSRK-Km for complementation | This study |
| pSRK-Km-*SmlsrB* | Coding region of *smc01225* in pSRK-Km for complementation | This study |
| pSRK-Km-*vrsA* | Coding region of *vrsA* in pSRK-Km for complementation | This study |
| prAtu2186 | Coding region of *atu2186* in pASK-IBA7 for recombinant protein purification | This study |
| rVtlR-IBA7 | Coding region of *bab1_1517* in pASK-IBA7 for recombinantly protein purification | [3] |
| prLsrB | Coding region of *smc01225* in pASK-IBA7 for recombinantly protein purification | This study |
| p*atu4670* | In-frame deletion of *atu4670* plus 1-kb of each flanking region in pNPTS138 | This study |

**References**

1. Wilms, I., Overloper, A., Nowrousian, M., Sharma, C. M. & Narberhaus, F. Deep sequencing uncovers numerous small RNAs on all four replicons of the plant pathogen *Agrobacterium tumefaciens*. *RNA Biol* **9**, 446-457, doi:10.4161/rna.17212 (2012).

2. Khan SR, Gaines J, Roop RM, 2nd, Farrand SK. Broad-host-range expression vectors with tightly regulated promoters and their use to examine the influence of TraR and TraM expression on Ti plasmid quorum sensing. Appl Environ Microbiol **74**, 5053-62 (2008).

3. Sheehan LM, Budnick JA, Blanchard C, Dunman PM, Caswell CC. A LysR-family transcriptional regulator required for virulence in *Brucella abortus* is highly conserved among the alpha-proteobacteria. Mol Microbiol doi:10.1111/mmi.13123 (2015).

**Images of northern blot analyses – Manuscript**


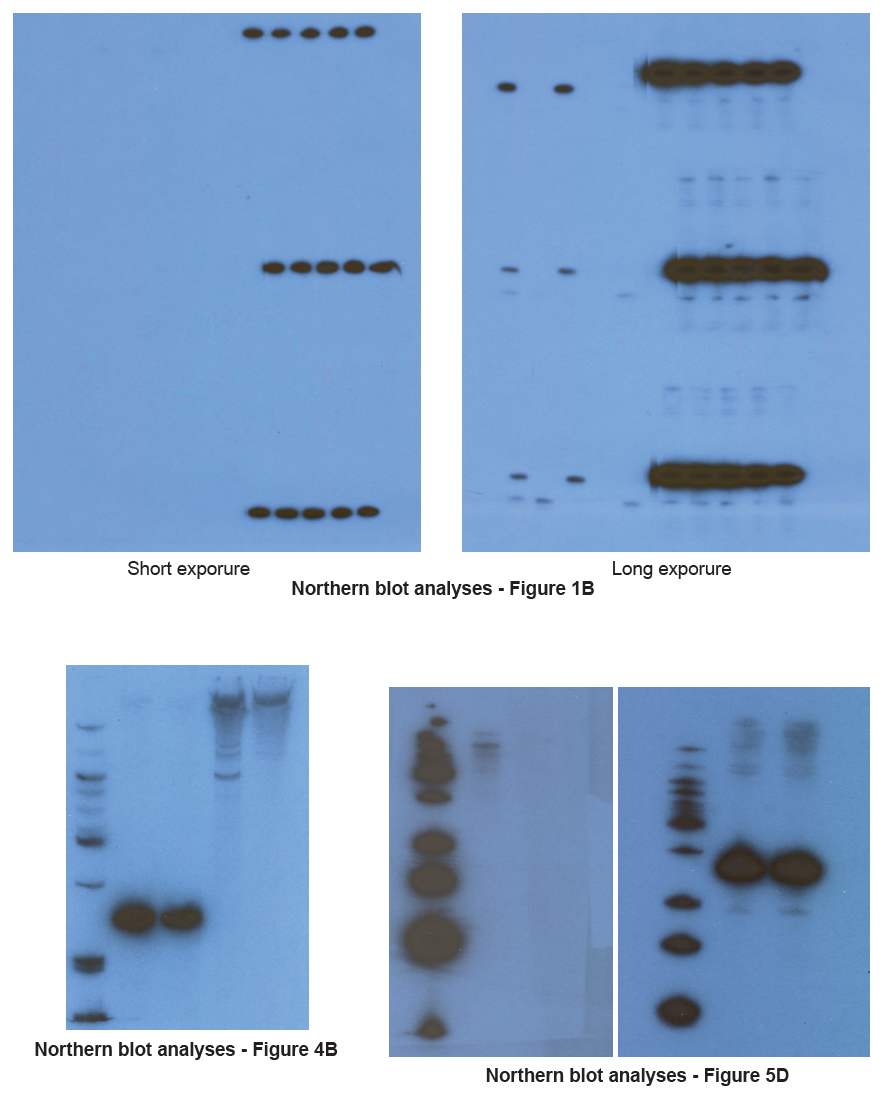


**Images of northern blot analyses – Figure S2**


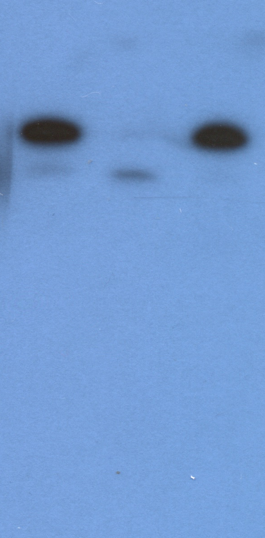


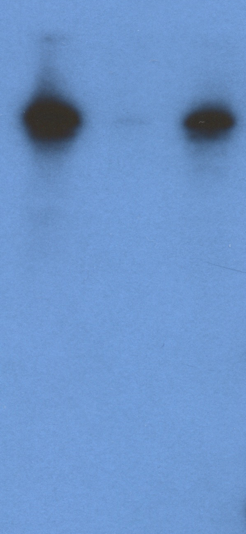


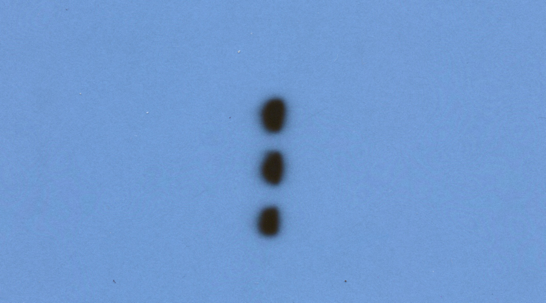


5S rRNA

AbcRs

VrsA


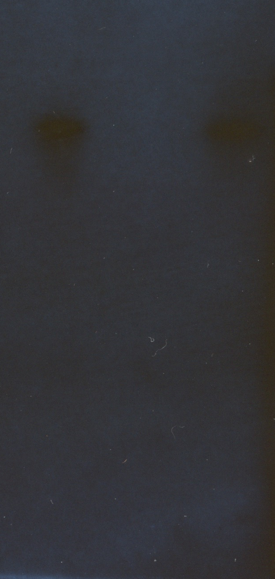

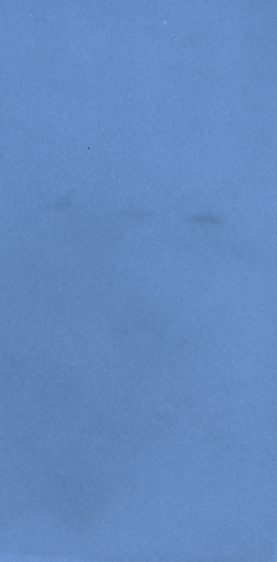


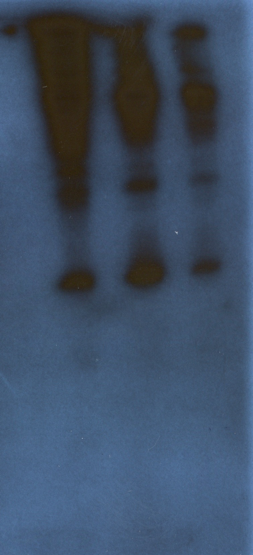


Atu0985-0986

Atu0612-0613

Atu0052-0053


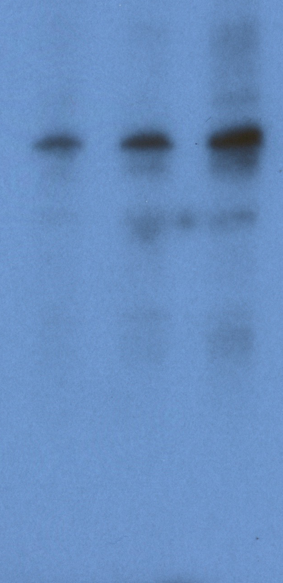

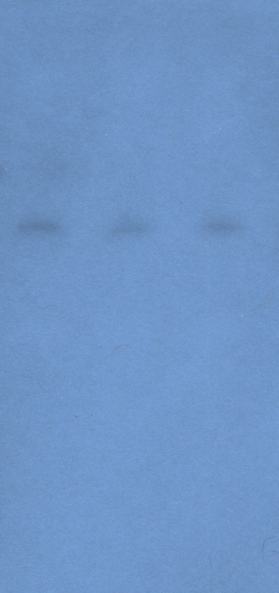


Atu2524


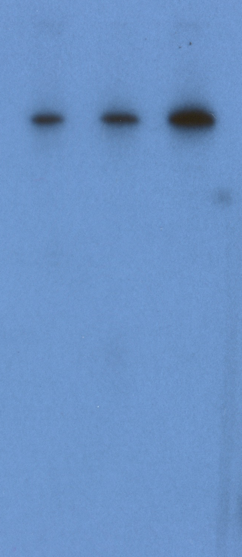


Atu1061-1062

Atu4671-4673

**Images of northern blot analyses – Figure S4**


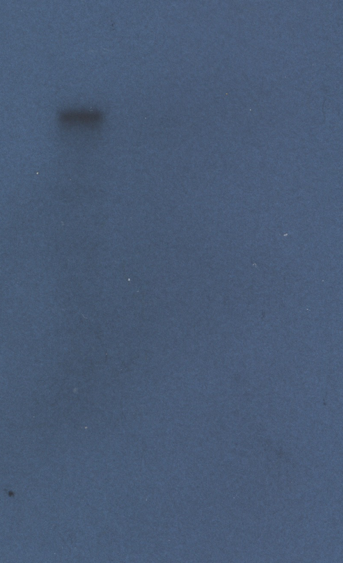

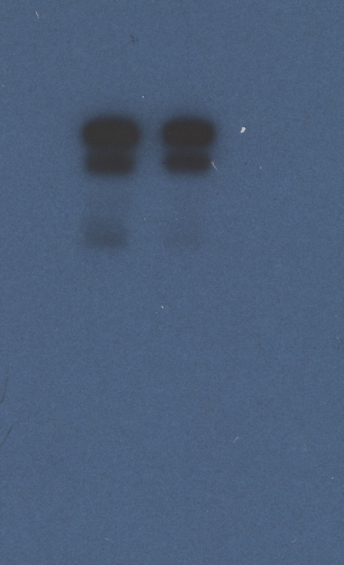


VrsA

AbcRs
